# Supplementary material for: Synergistic effect of inhibiting CHK2 and DNA replication on cancer cell growth
Source: eLife. 2025 Jan 31;13:RP104718. doi: 10.7554/eLife.104718 (PMC11785374; doi:10.7554/eLife.104718)
Supplement: Figure 2—source data 1. — 4 and 7 are our codes for BKC and IBC, respectively. d: DMSO control; 7 4: means BKC + IBC. [file elife-104718-fig2-data1.zip › Figure 2-Source Data-1.pdf]

Phospho-CHK1 (S354)

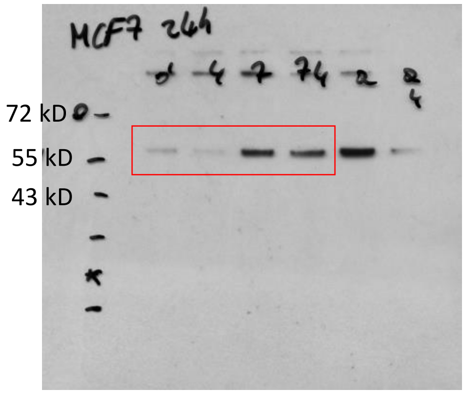

Total CHK1

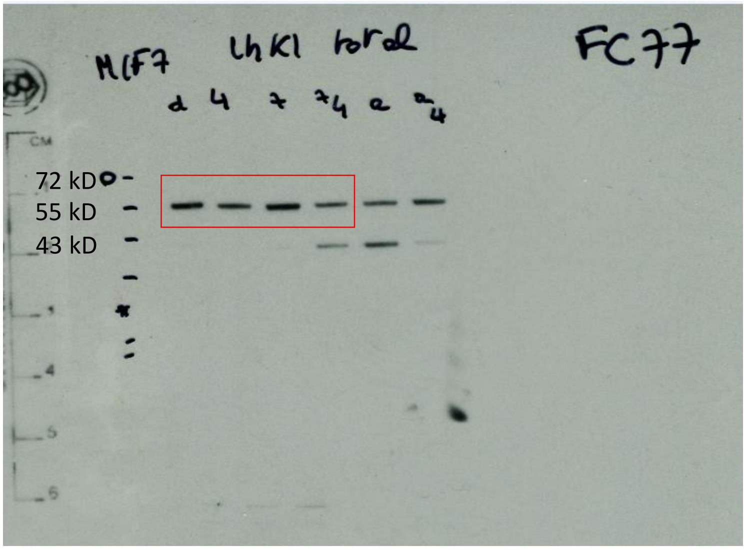

Figure 2, Sources Data 1. Original membranes corresponding to Figure 2C. 4 and 7 are our codes BKC and IBC, respectively. d: DMSO control; 7 4: means BKC+IBC.

Figure 2. IBC and BKC induce replication stress and impede fork progression. C. MCF-7 cells were treated with IBC/BKC for 24 hours, as in panel A, and CHK1 phosphorylated on S345 (pCHK1) was detected by western blotting. The ratio of pCHK1 to total CHK1, relative to the DMSO control, is indicated. A representative example of two independent experiments is shown.
