# Supplementary figures and images for: Synergistic effect of inhibiting CHK2 and DNA replication on cancer cell growth
Source: eLife. 2025 Jan 31;13:RP104718. doi: 10.7554/eLife.104718 (PMC11785374; doi:10.7554/eLife.104718)

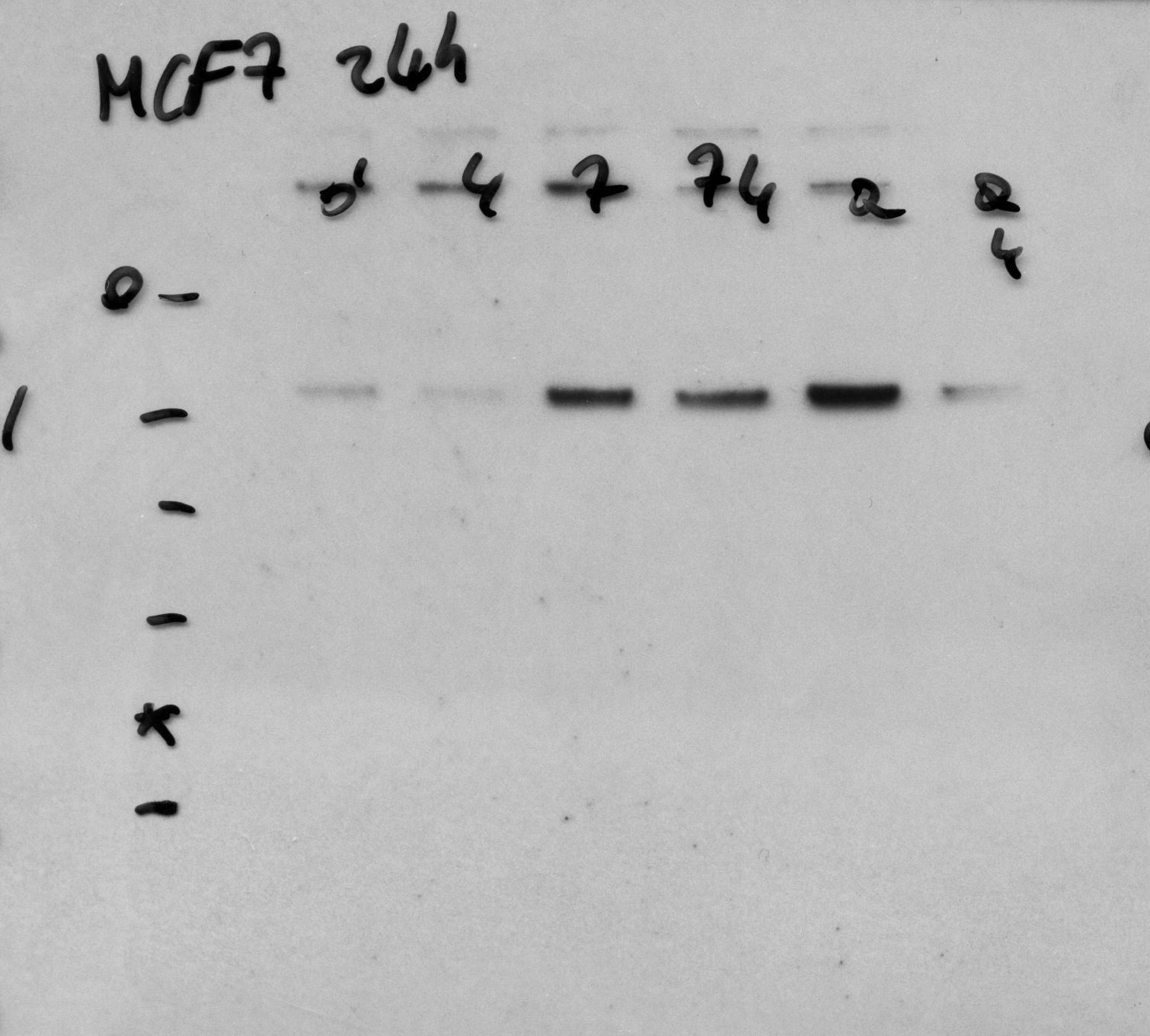

Supplement: Figure 2—source data 2. [file elife-104718-fig2-data2.zip › Fig.2C-1.tif]

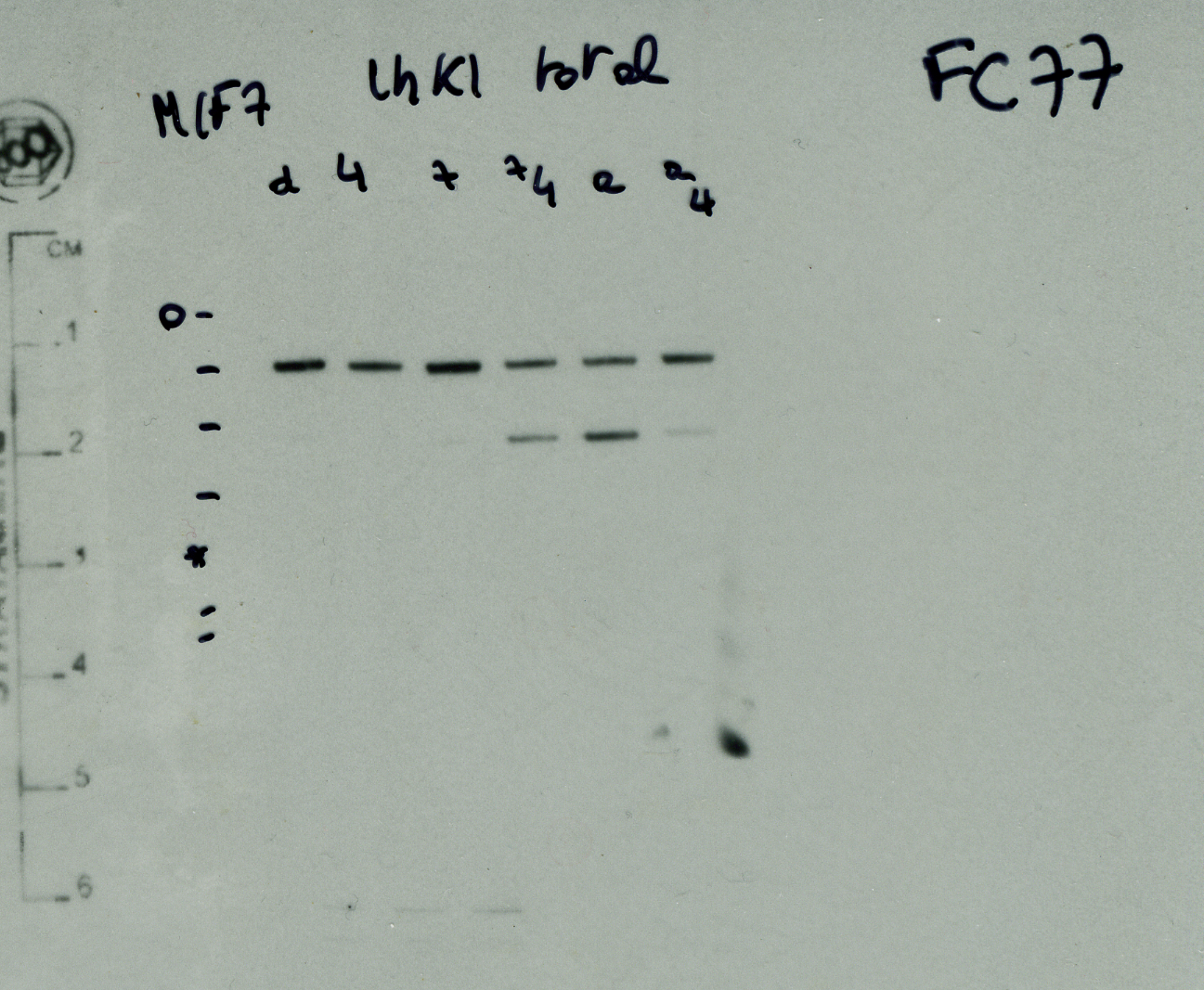

Supplement: Figure 2—source data 2. [file elife-104718-fig2-data2.zip › Fi.2C-2.tif]

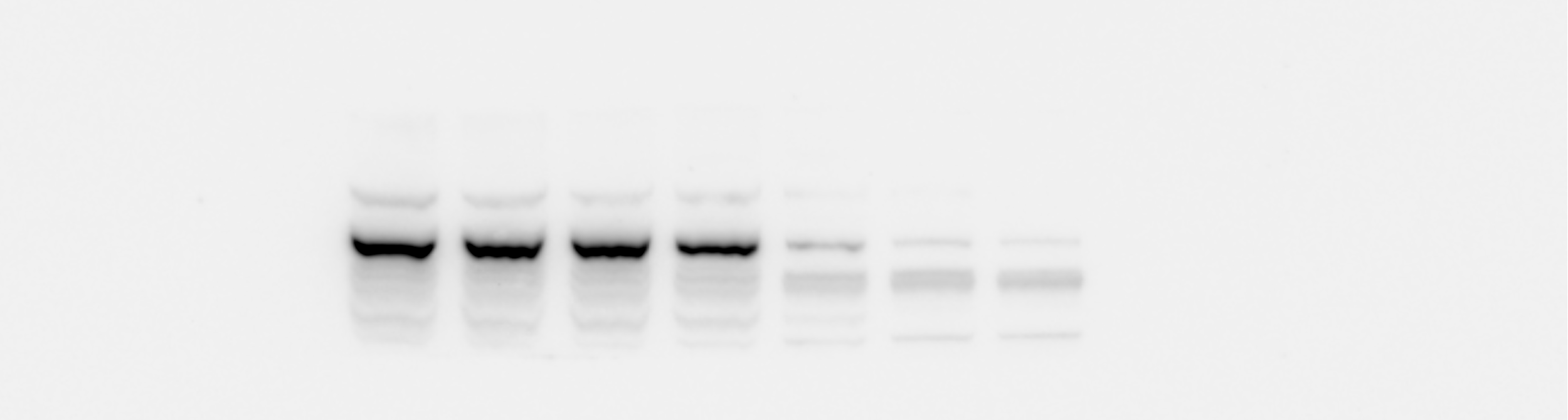

Supplement: Figure 3—source data 2. [file elife-104718-fig3-data2.zip › Aphidicolin.tif]

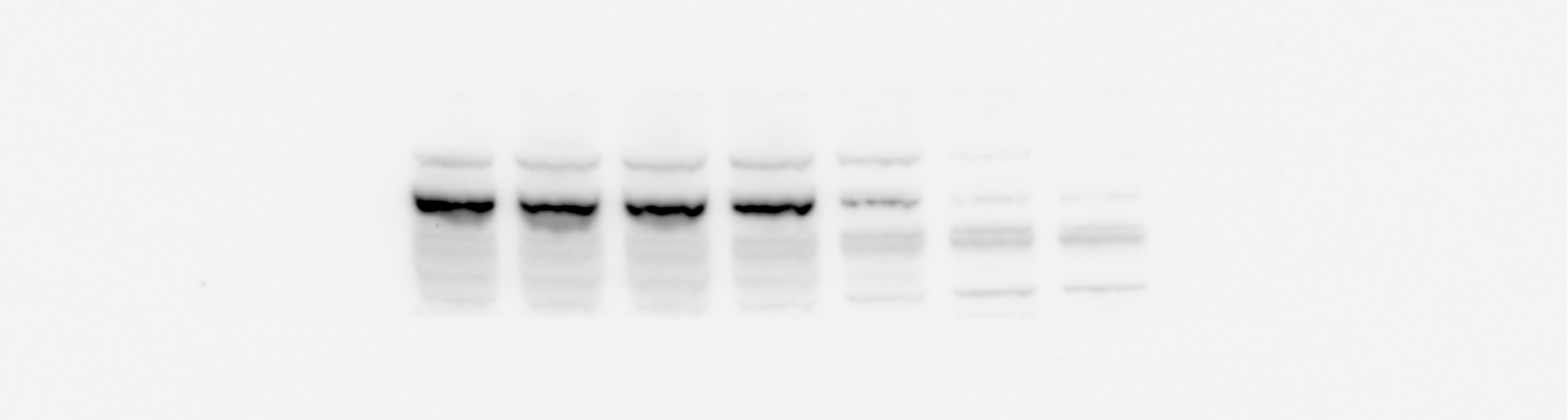

Supplement: Figure 3—source data 2. [file elife-104718-fig3-data2.zip › BKC.tif]

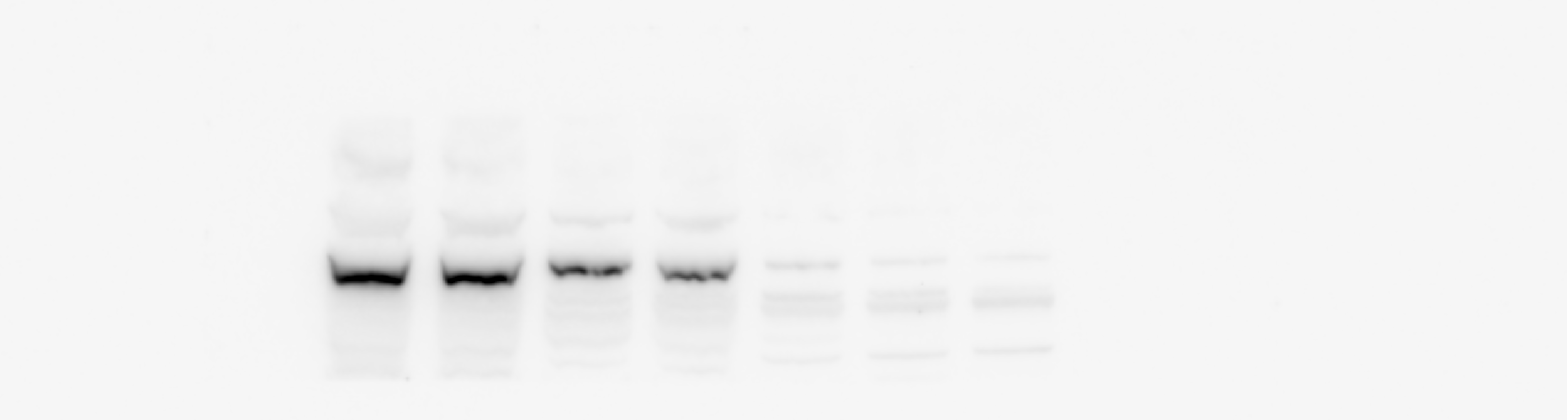

Supplement: Figure 3—source data 2. [file elife-104718-fig3-data2.zip › DMSO.tif]

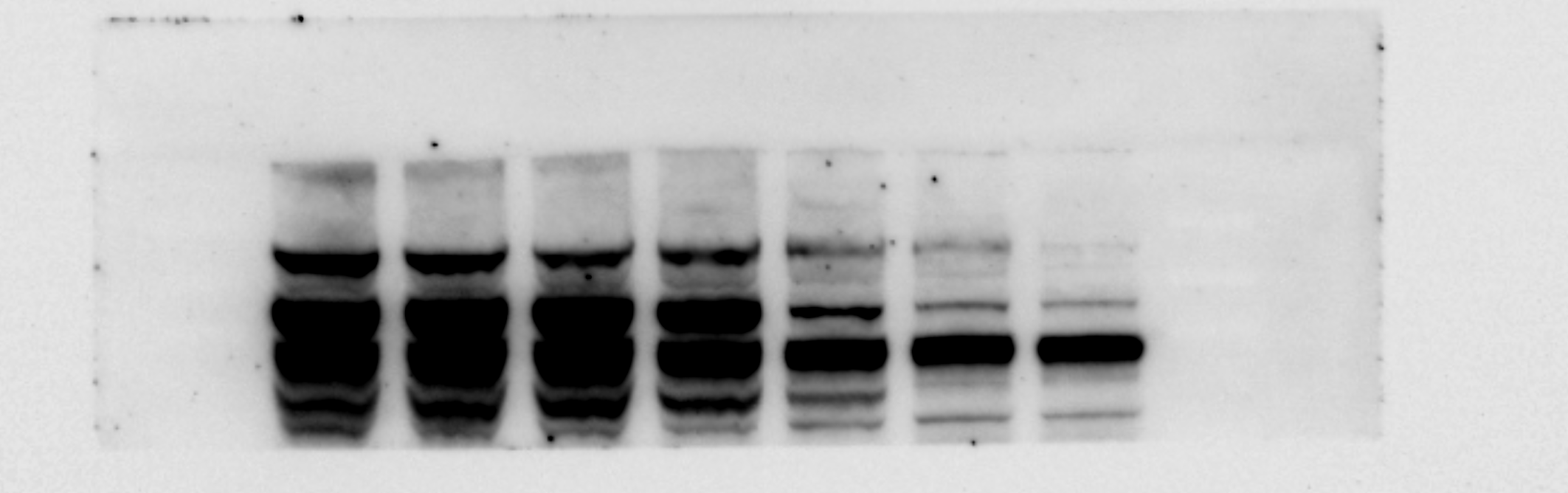

Supplement: Figure 3—source data 2. [file elife-104718-fig3-data2.zip › Aphidicolin-PolE.tif]

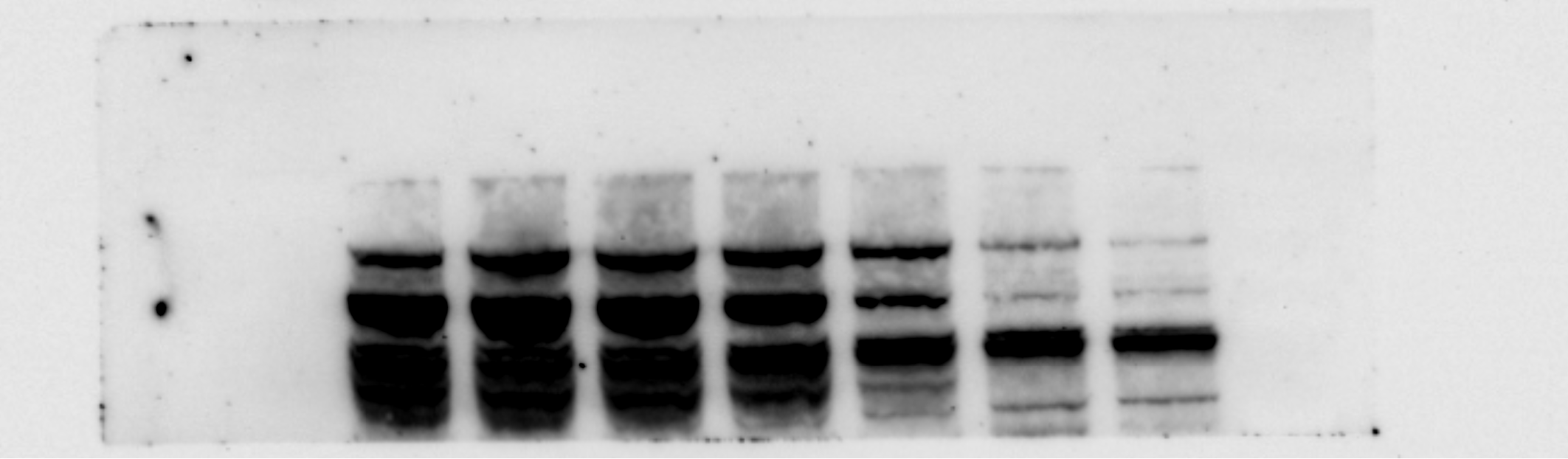

Supplement: Figure 3—source data 2. [file elife-104718-fig3-data2.zip › BKC-PolE.tif]

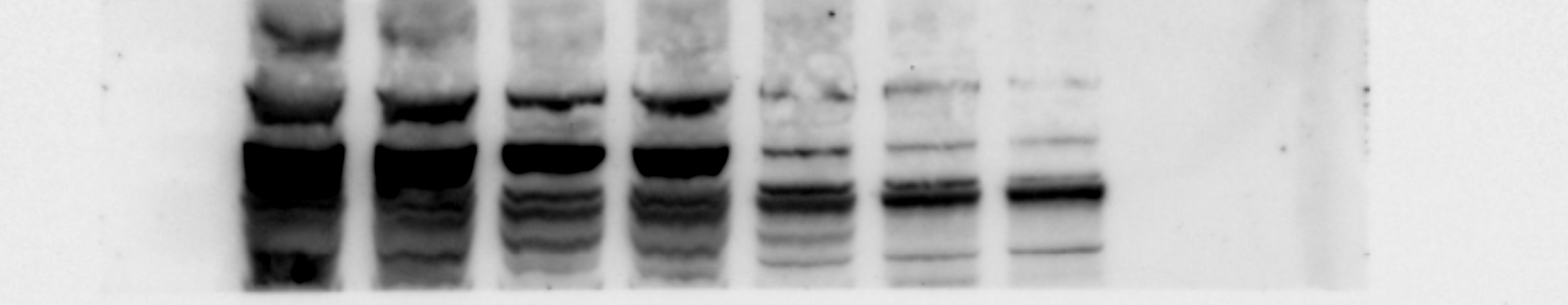

Supplement: Figure 3—source data 2. [file elife-104718-fig3-data2.zip › DMSO-PolE.tif]

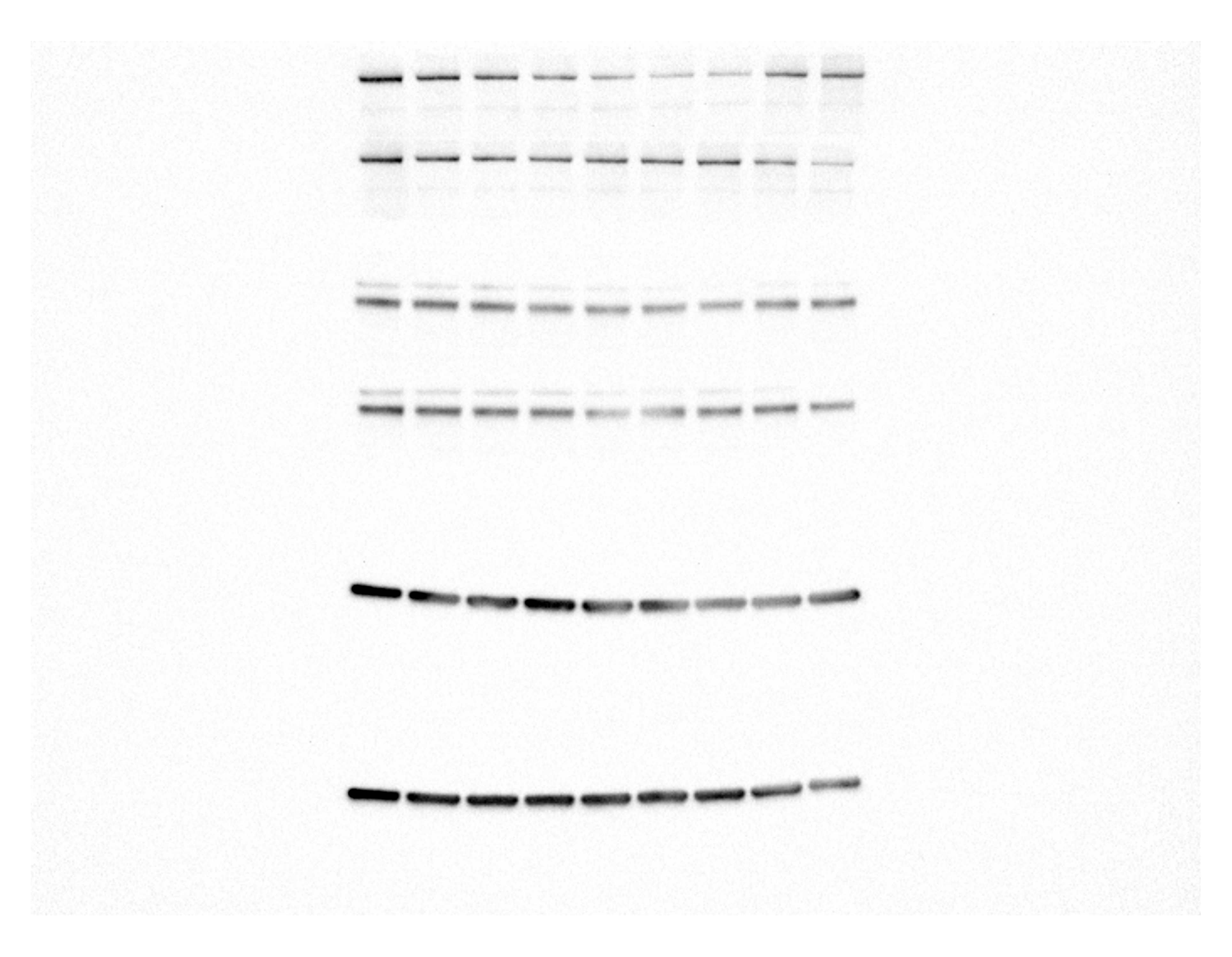

Supplement: Figure 3—figure supplement 1—source data 2. [file elife-104718-fig3-figsupp1-data2.zip › Figure 3-figure sapplement 1-source data 2.tif]

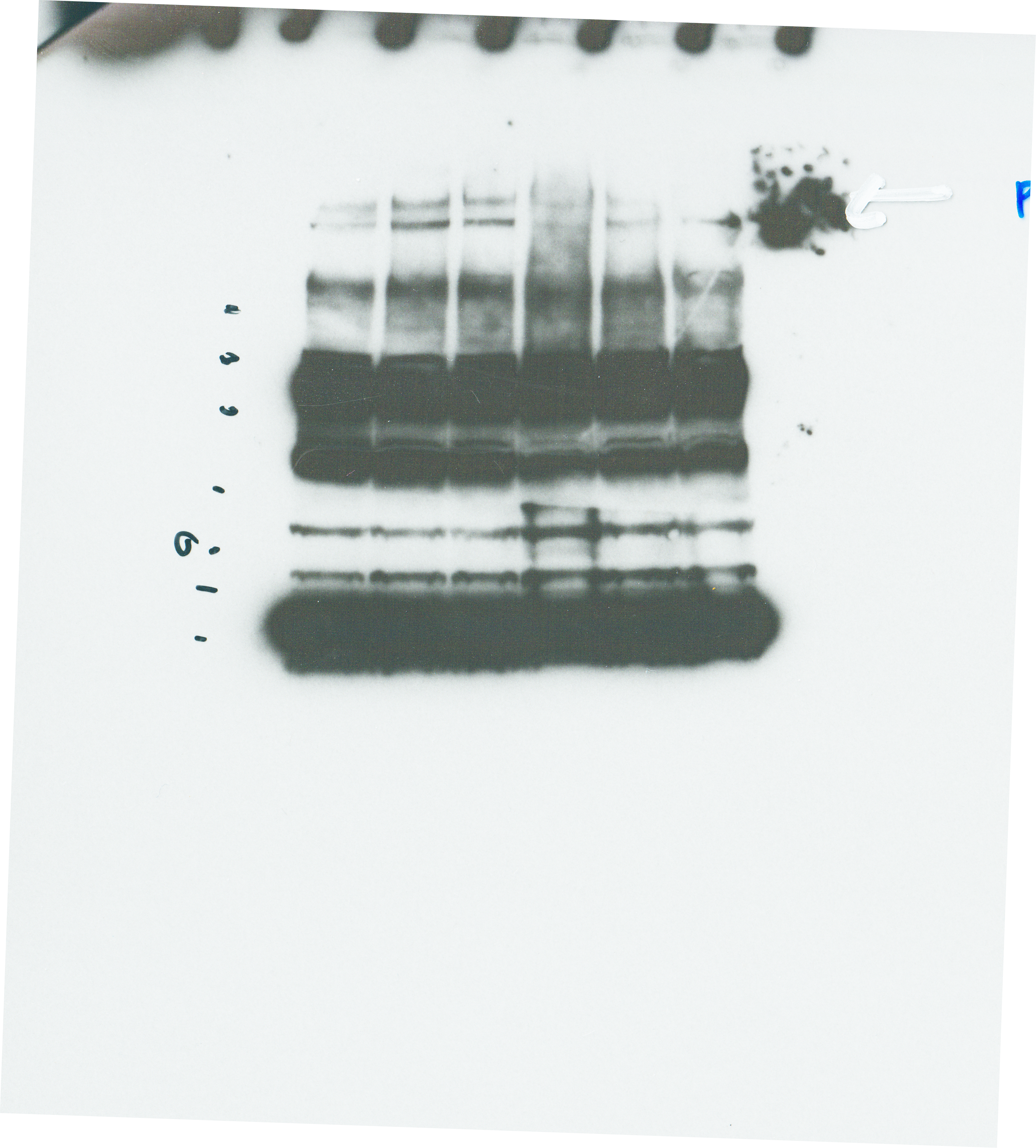

Supplement: Figure 4—source data 2. [file elife-104718-fig4-data2.zip › pBRCA1-S988.tiff]

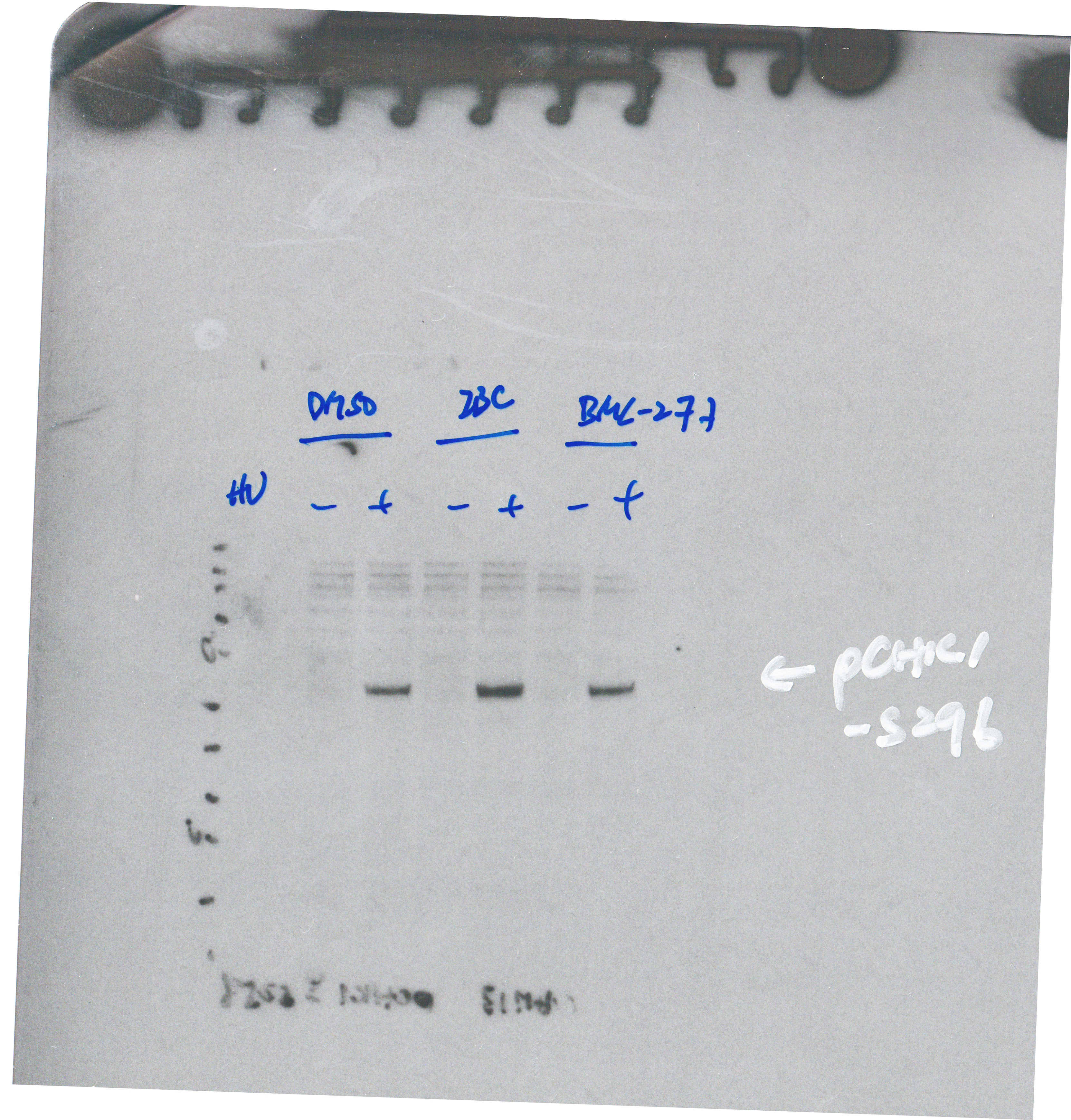

Supplement: Figure 4—source data 2. [file elife-104718-fig4-data2.zip › pCHK1-S296.tiff]

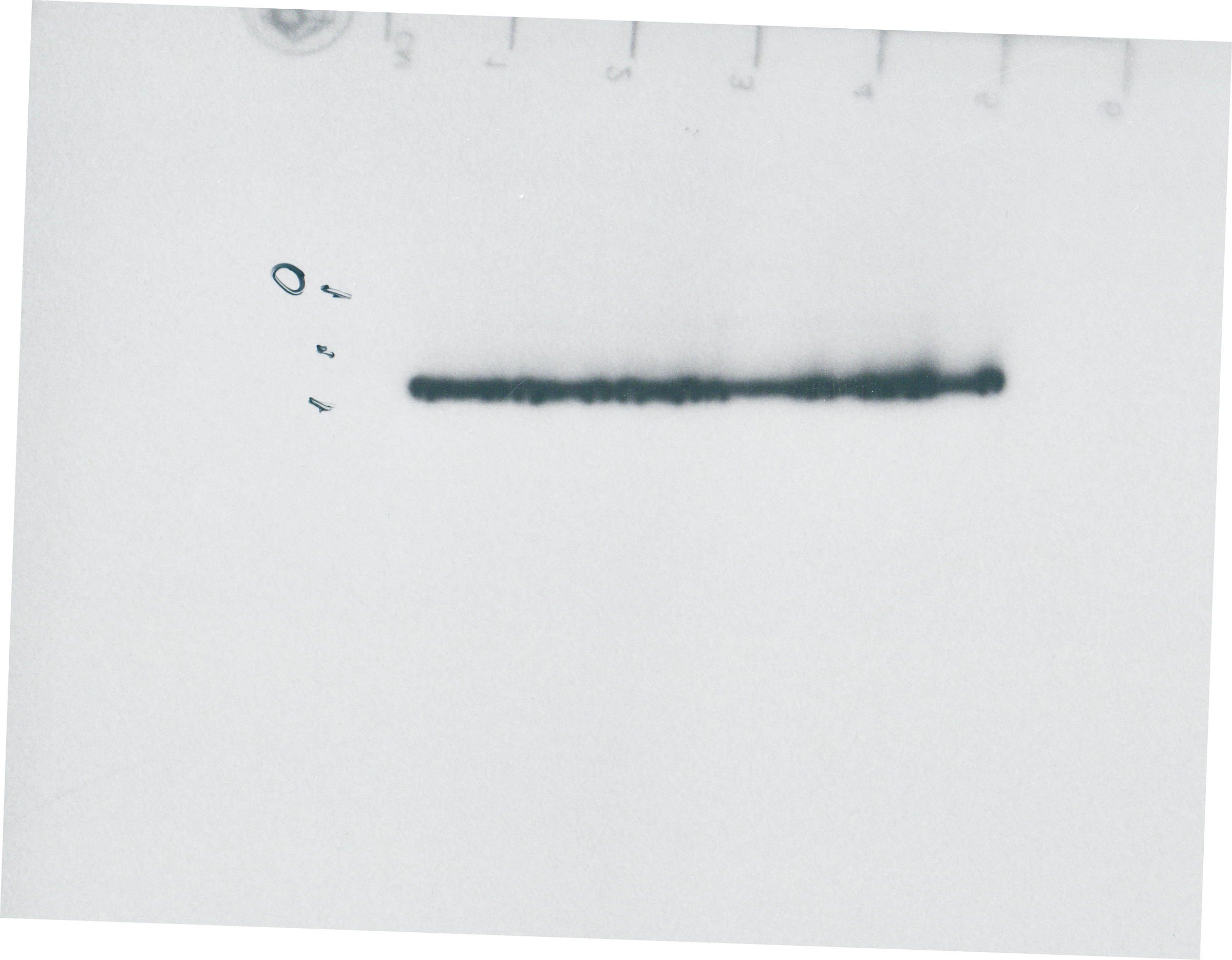

Supplement: Figure 4—source data 2. [file elife-104718-fig4-data2.zip › TBP.tiff]

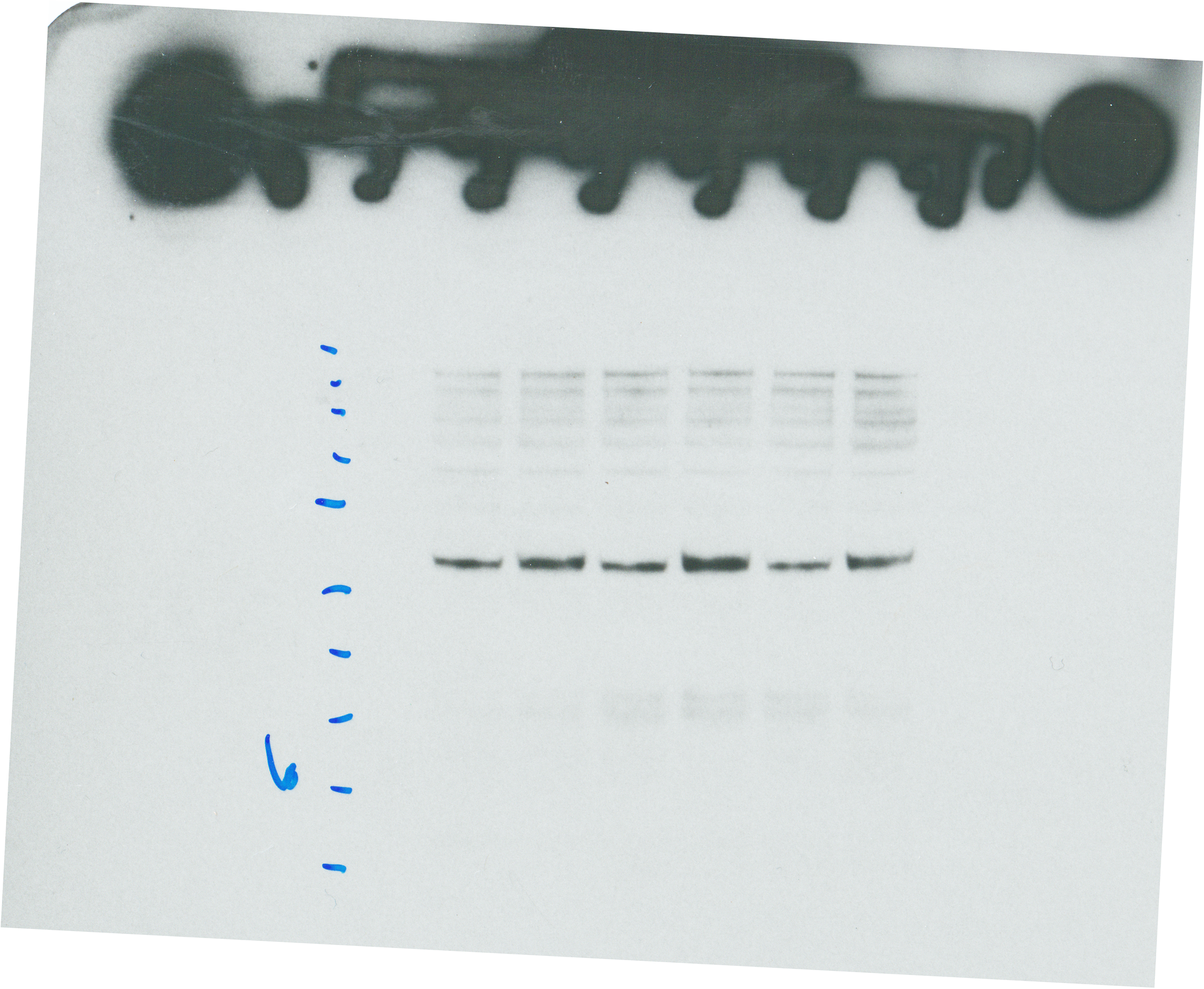

Supplement: Figure 4—source data 2. [file elife-104718-fig4-data2.zip › total CHK1.tiff]

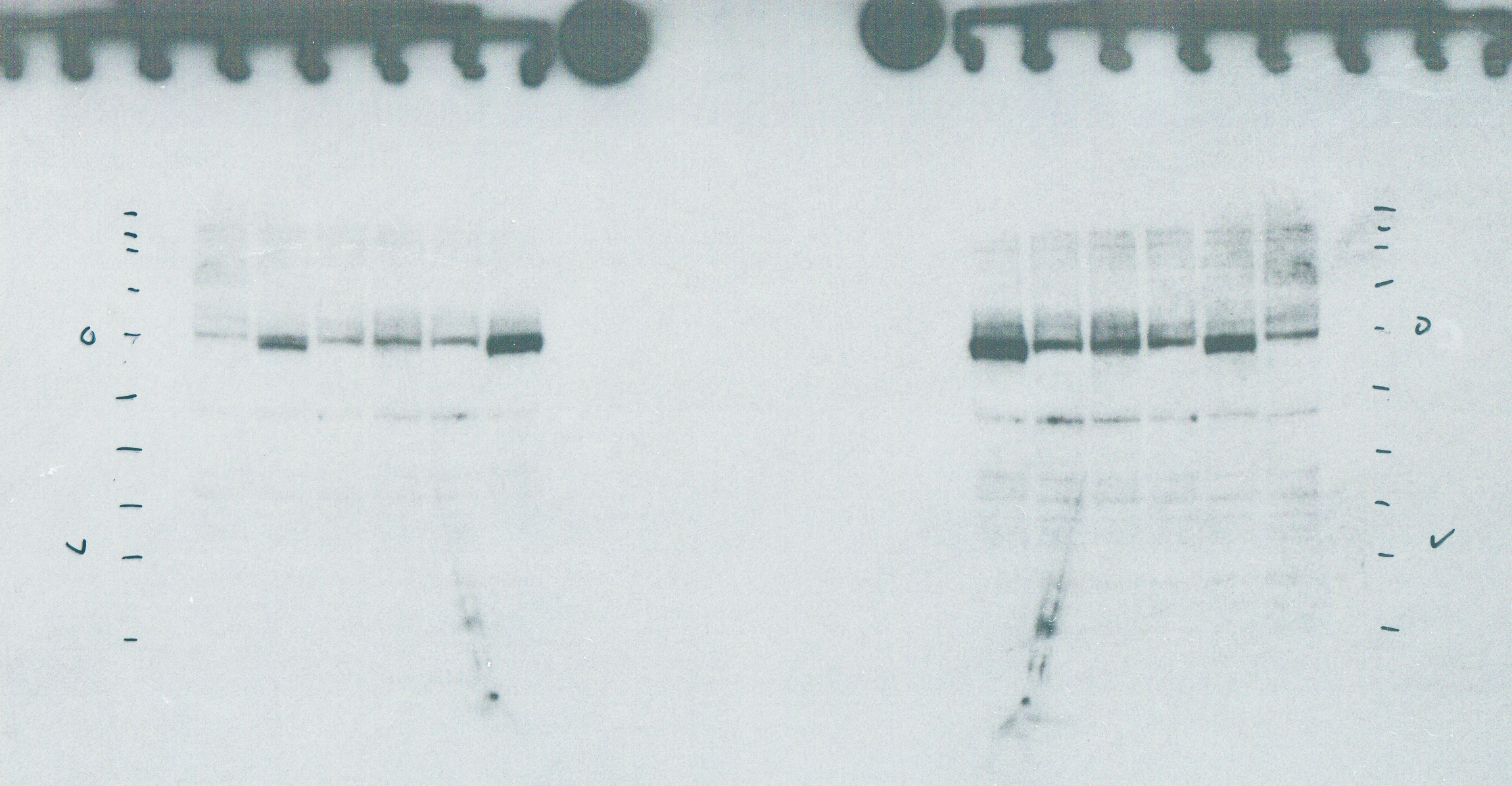

Supplement: Figure 4—source data 2. [file elife-104718-fig4-data2.zip › pCHK2-S516.tiff]

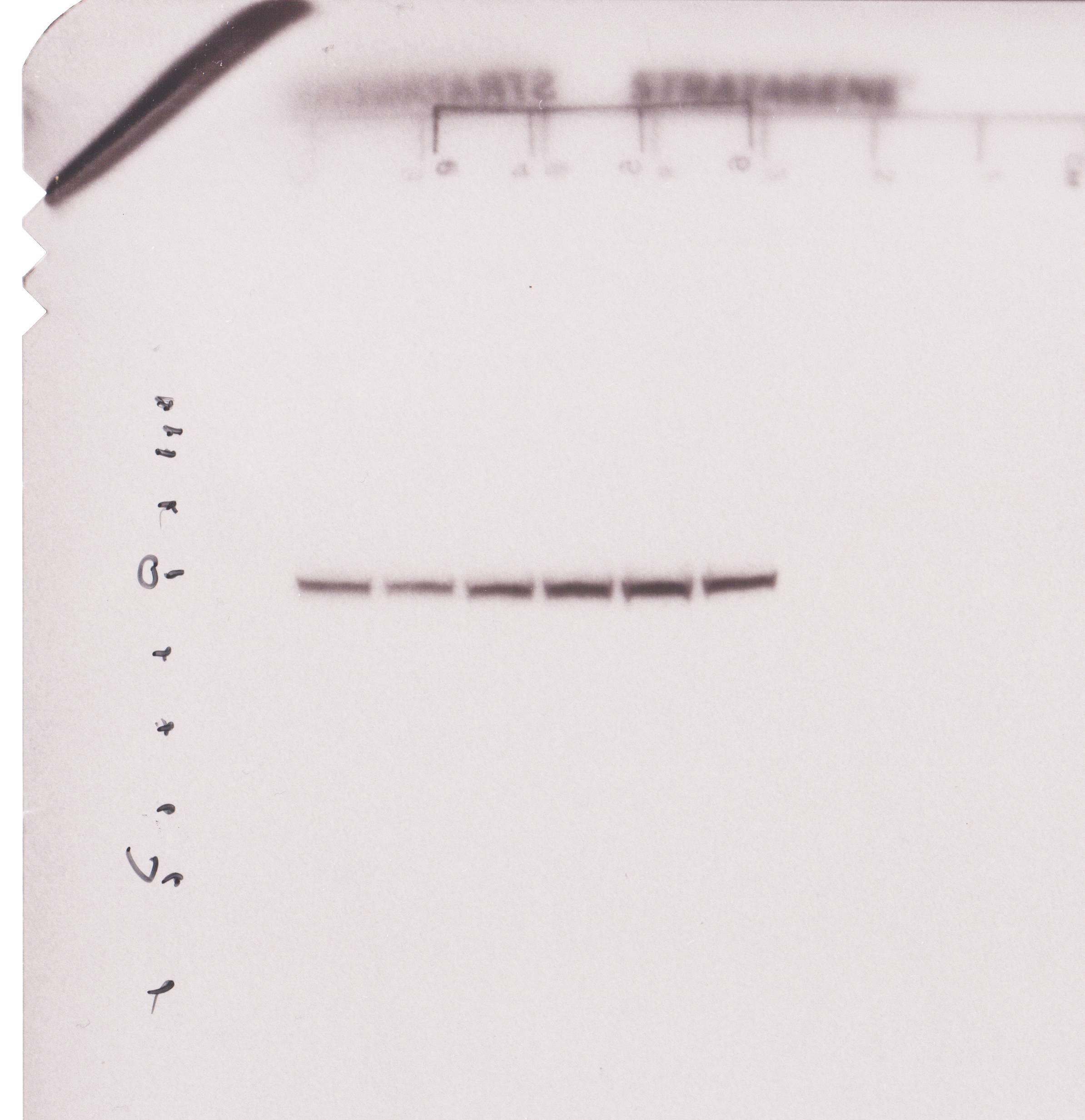

Supplement: Figure 4—source data 2. [file elife-104718-fig4-data2.zip › total CHK2.tiff]

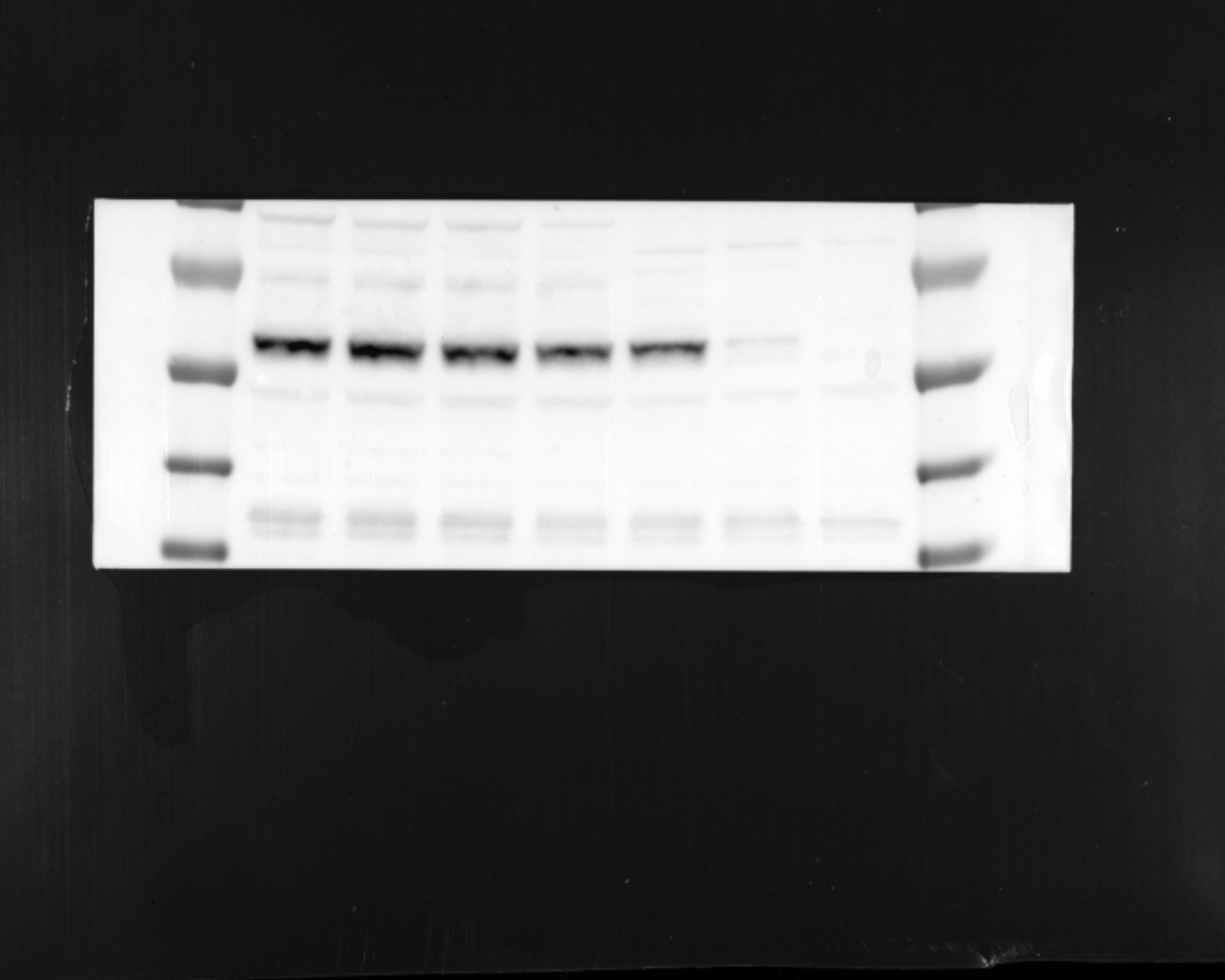

Supplement: Figure 4—source data 2. [file elife-104718-fig4-data2.zip › CHK1_CHEK2i+Ladder.tif]

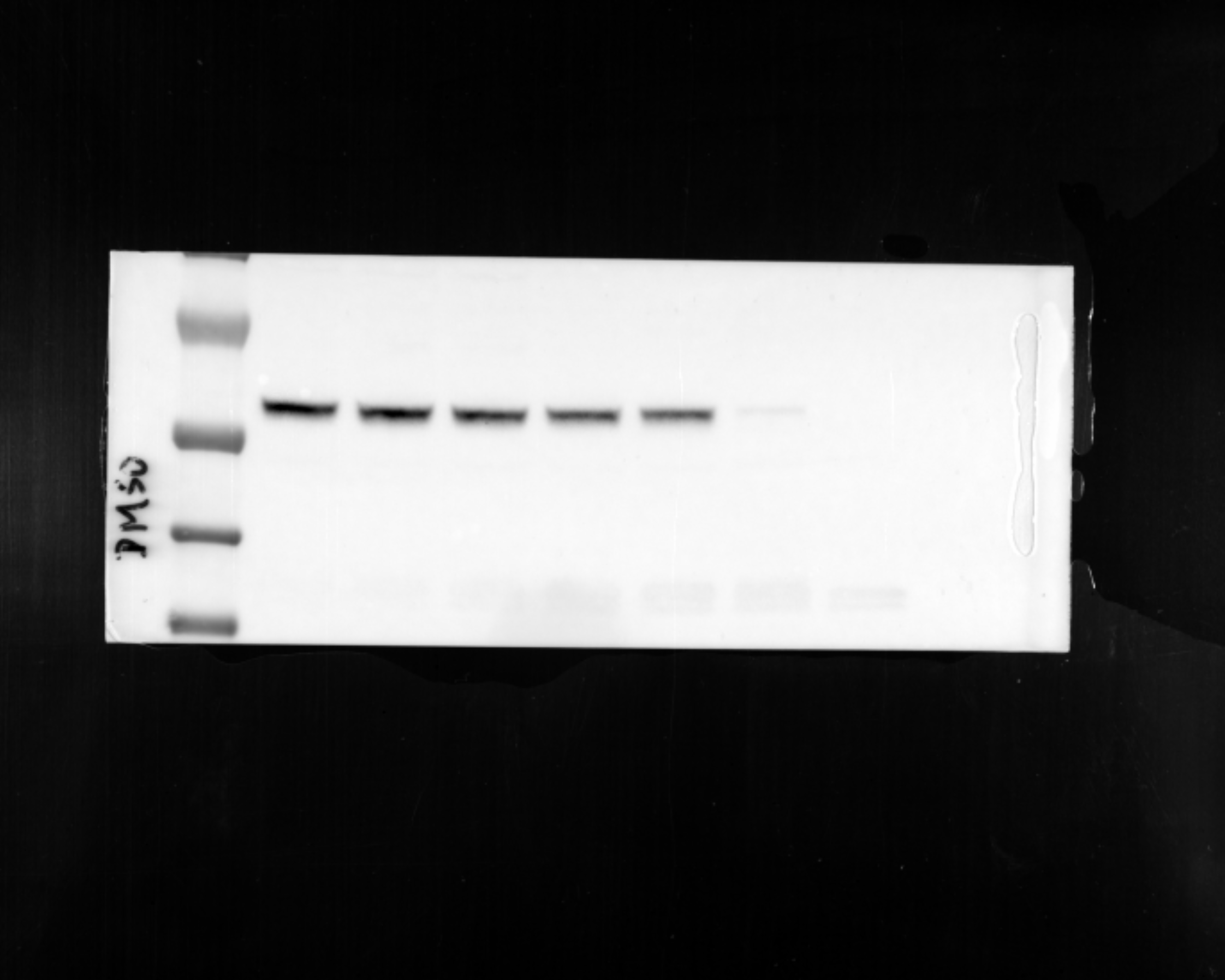

Supplement: Figure 4—source data 2. [file elife-104718-fig4-data2.zip › CHK1_DMSO+Ladder.tif]

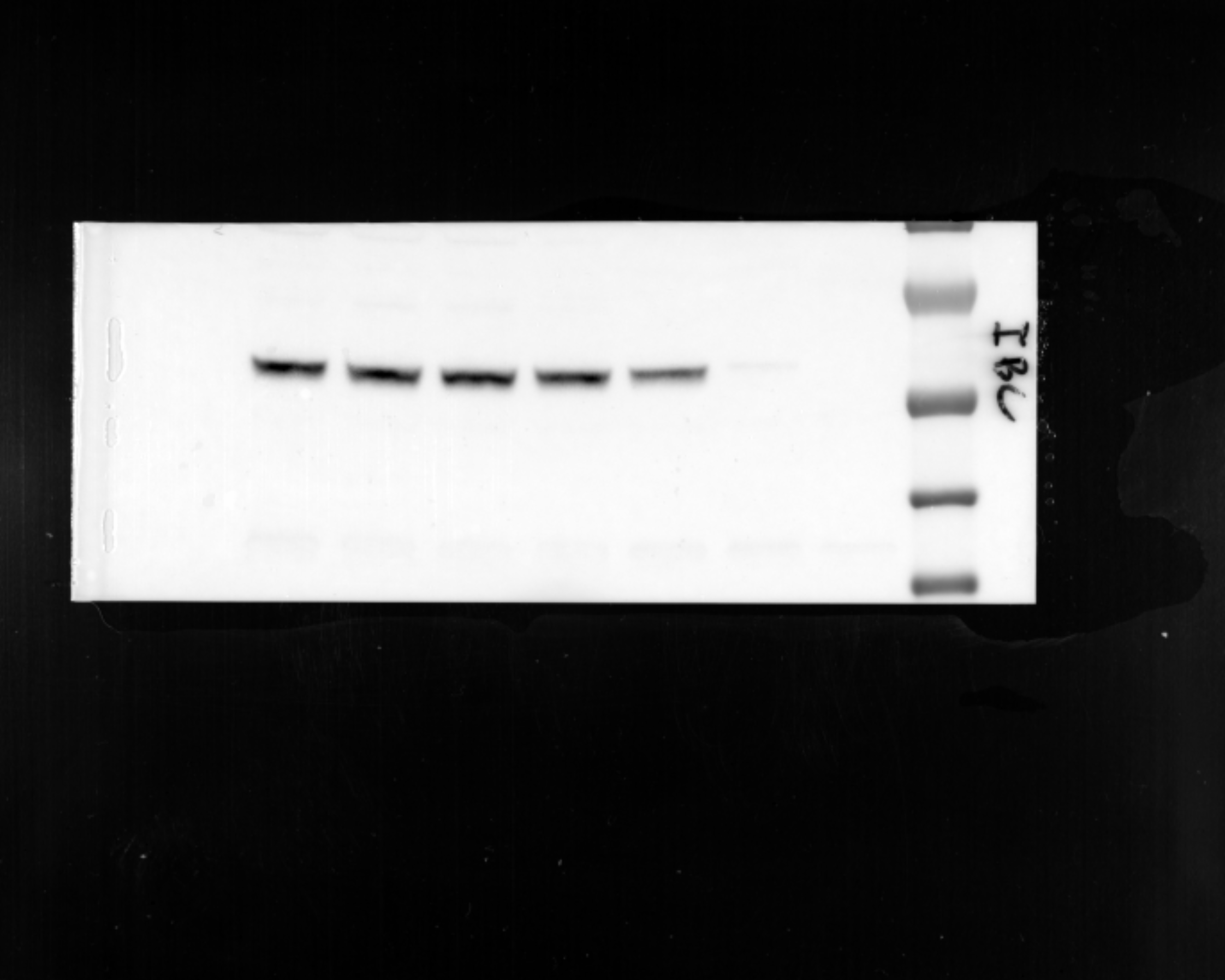

Supplement: Figure 4—source data 2. [file elife-104718-fig4-data2.zip › CHK1_IBC+Ladder.tif]

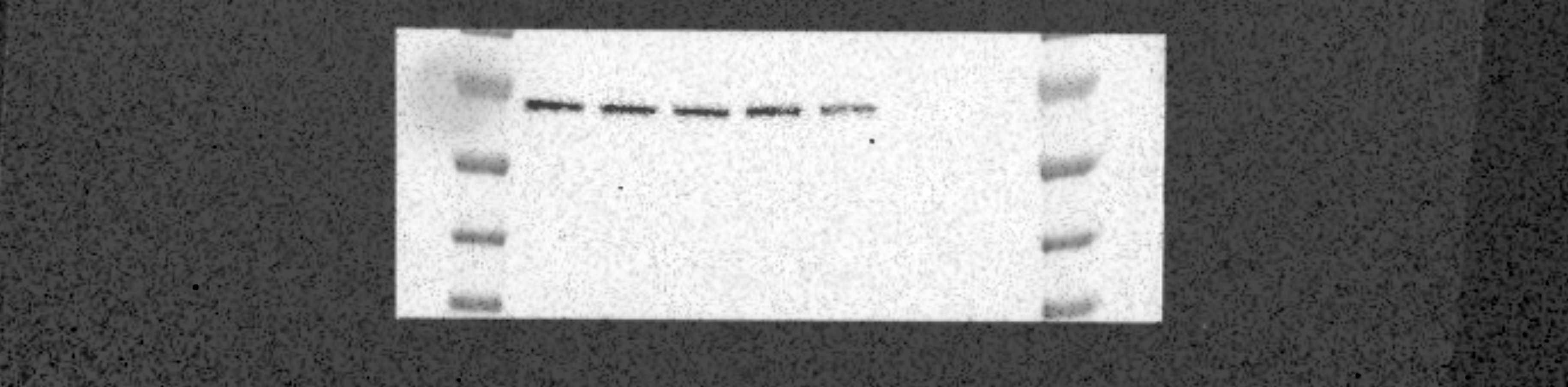

Supplement: Figure 4—source data 2. [file elife-104718-fig4-data2.zip › CHK2_CHEK2i+Ladder.tif]

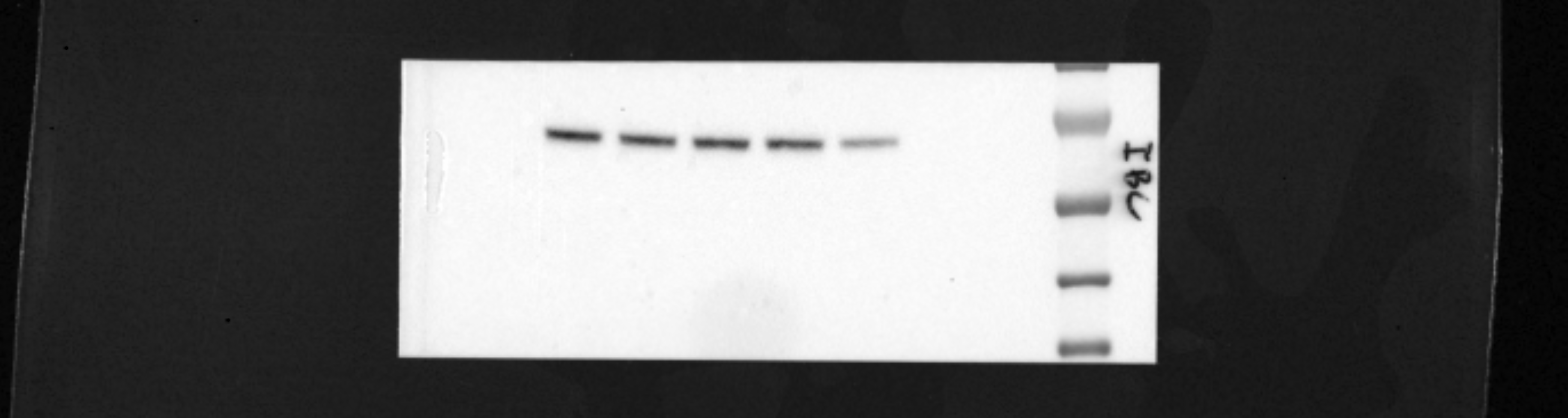

Supplement: Figure 4—source data 2. [file elife-104718-fig4-data2.zip › CHK2_IBC+Ladder.tif]

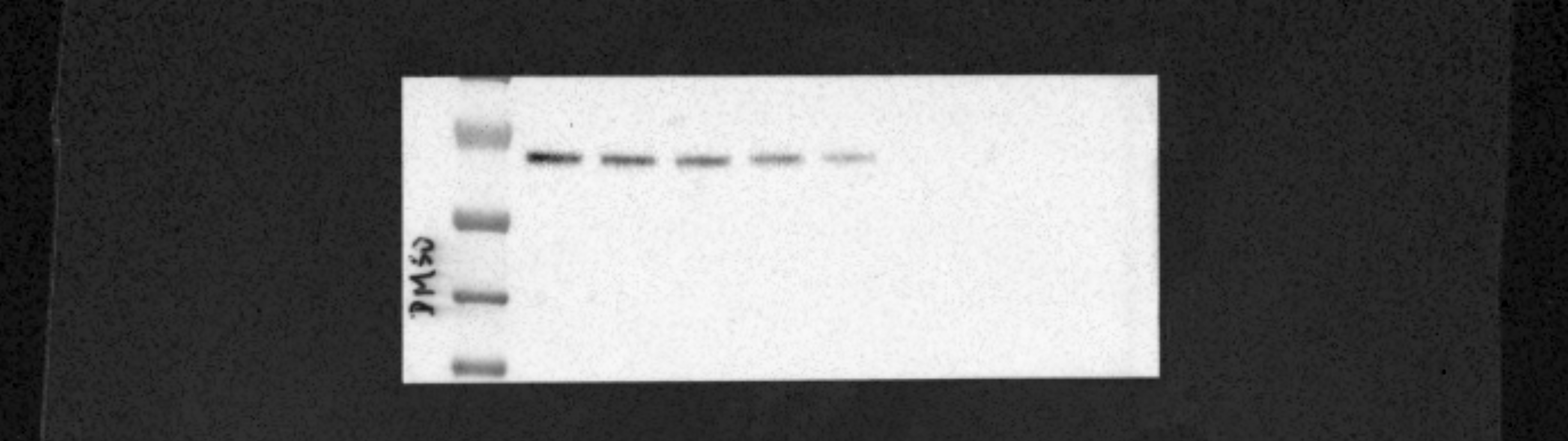

Supplement: Figure 4—source data 2. [file elife-104718-fig4-data2.zip › CHK2_DMSO+Ladder.tif]

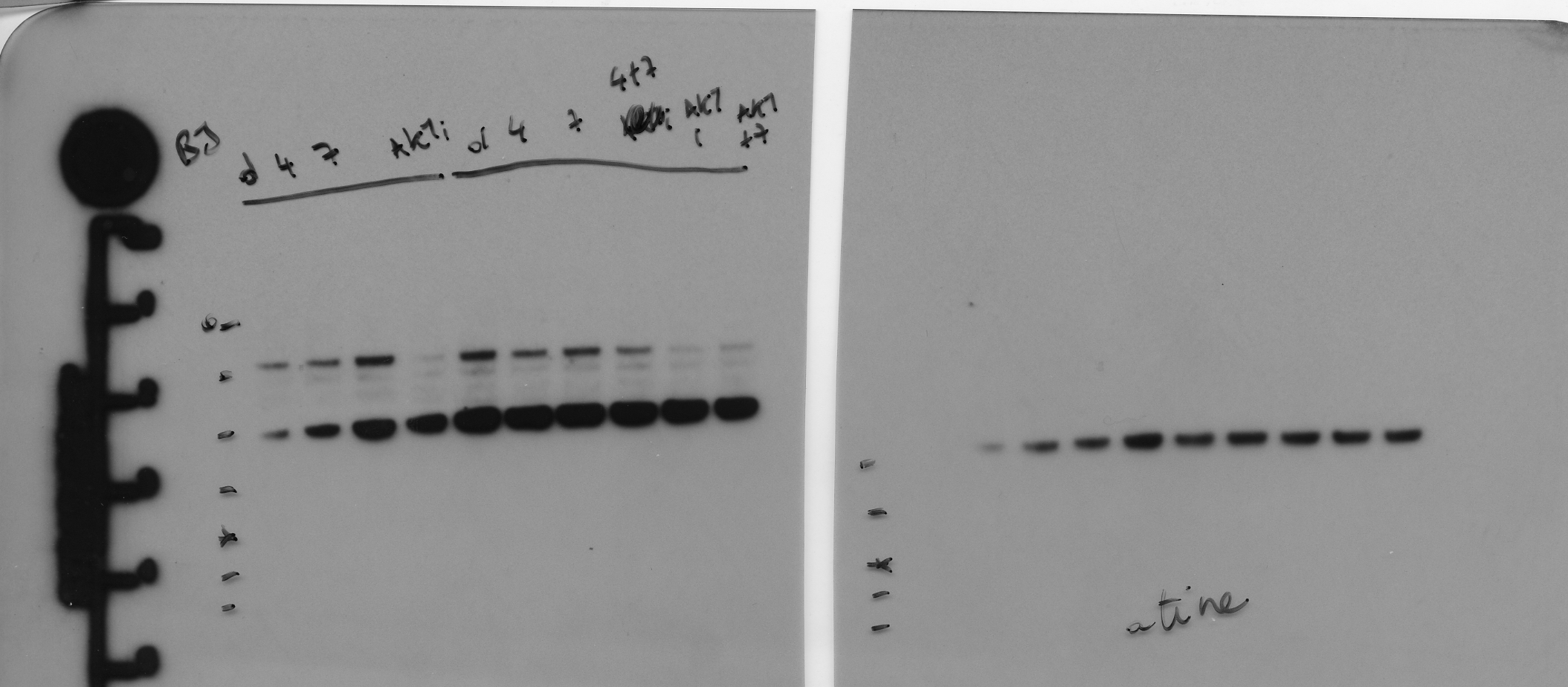

Supplement: Figure 4—figure supplement 1—source data 2. [file elife-104718-fig4-figsupp1-data2.zip › Figure 4-figure supplement 1-Source data 2.tif]

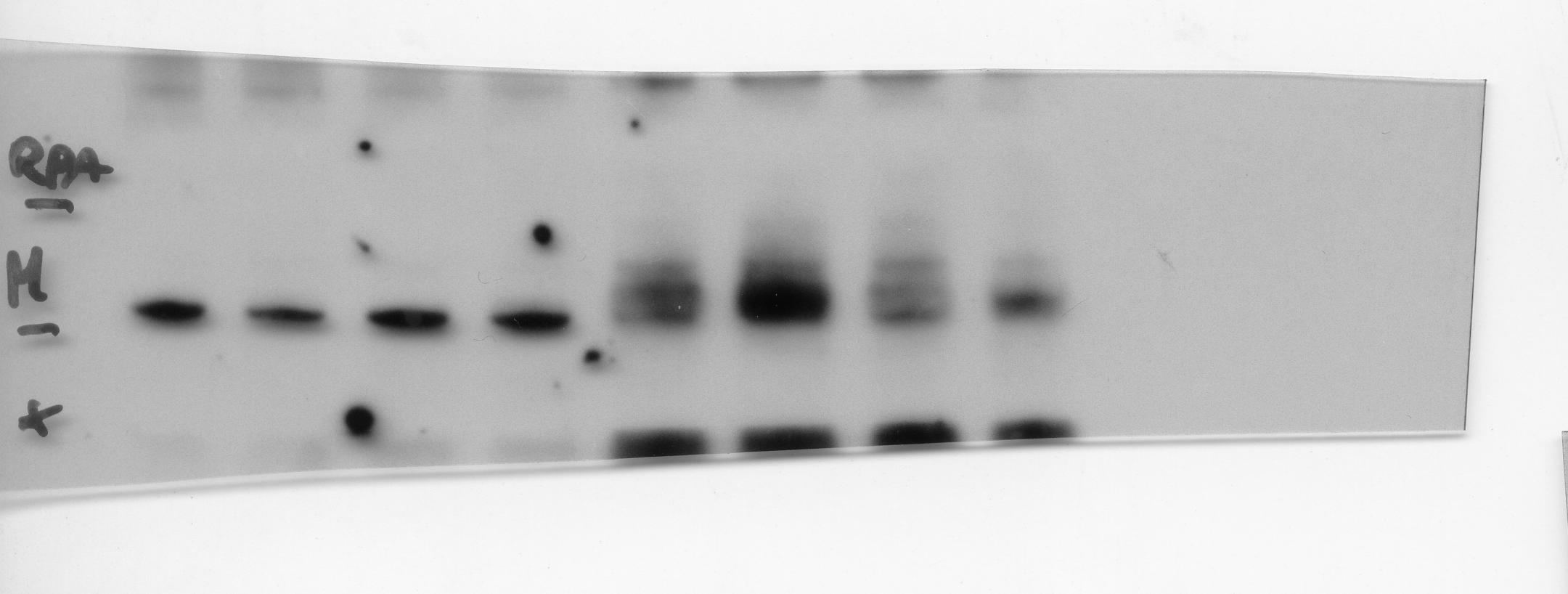

Supplement: Figure 5—source data 2. [file elife-104718-fig5-data2.zip › Fig.5C-1.tif]

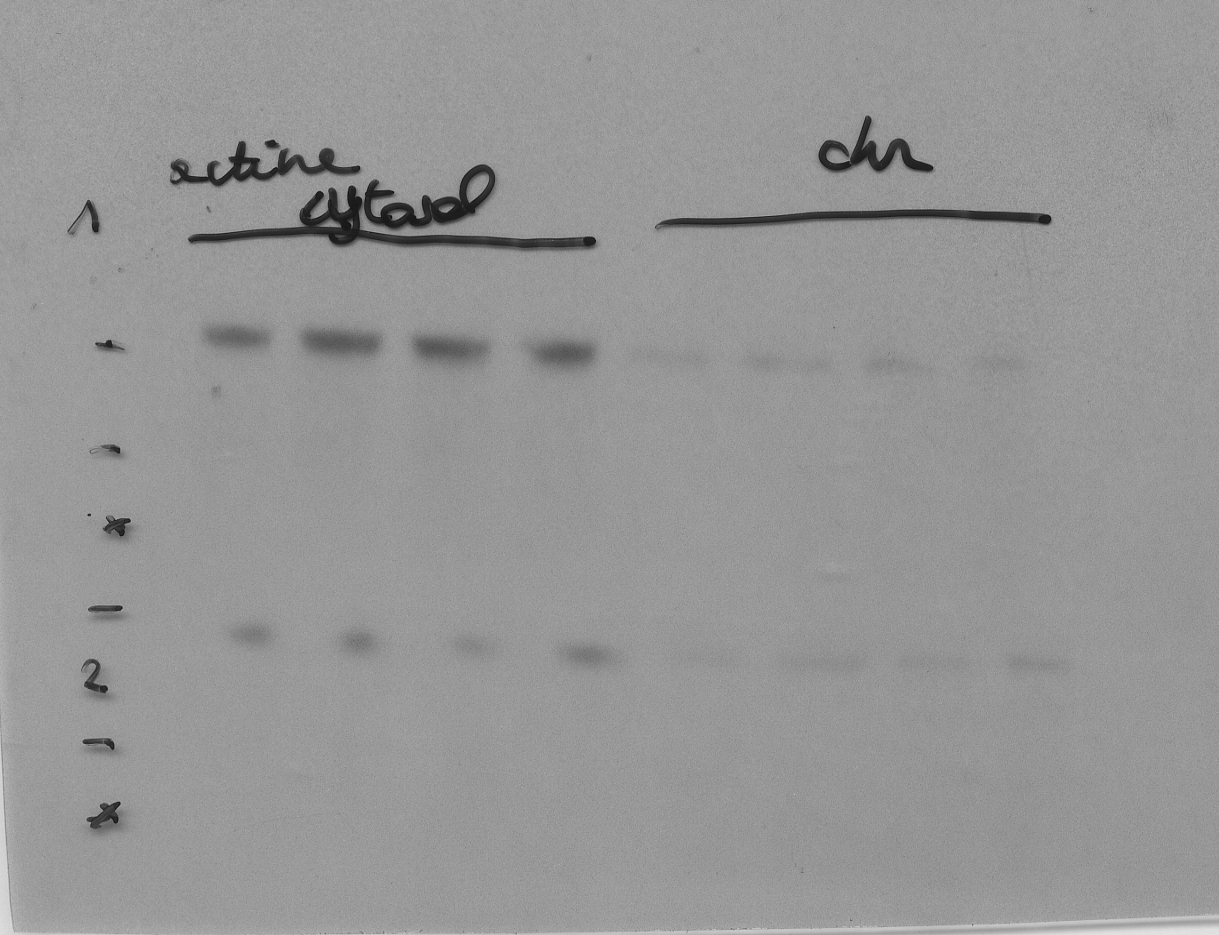

Supplement: Figure 5—source data 2. [file elife-104718-fig5-data2.zip › Fig.5C-2.tif]

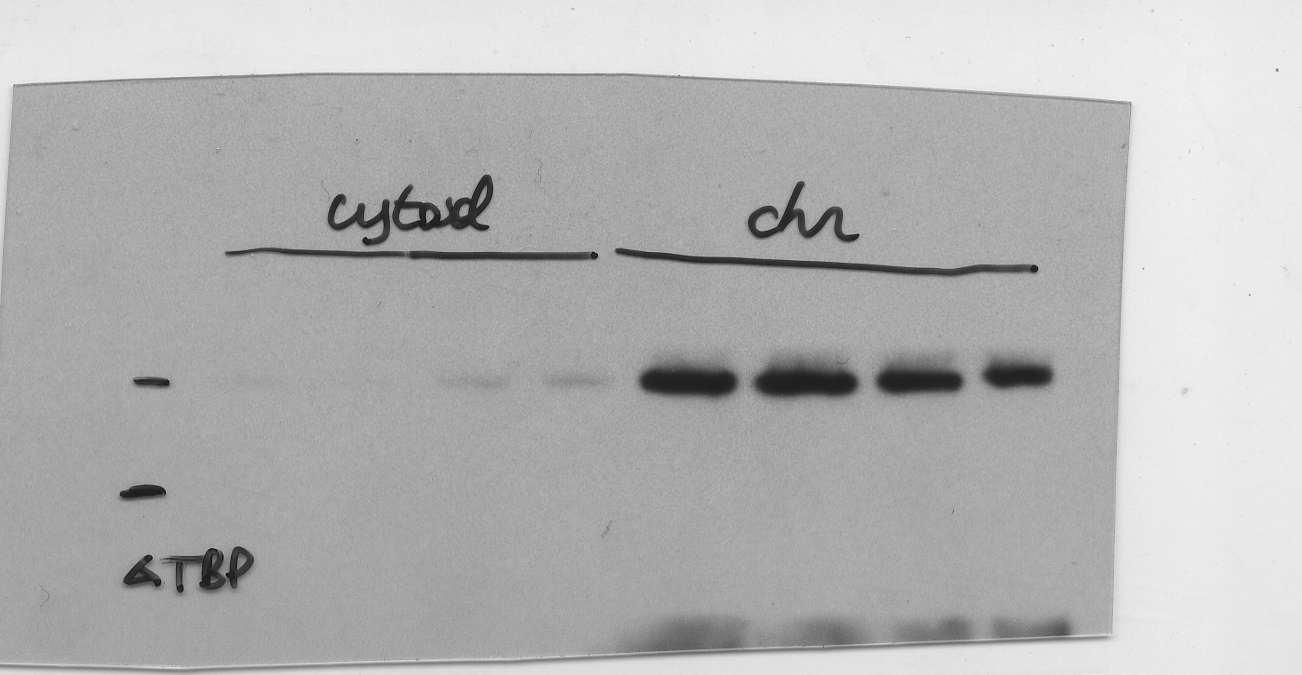

Supplement: Figure 5—source data 2. [file elife-104718-fig5-data2.zip › Fig.5C-3.tif]

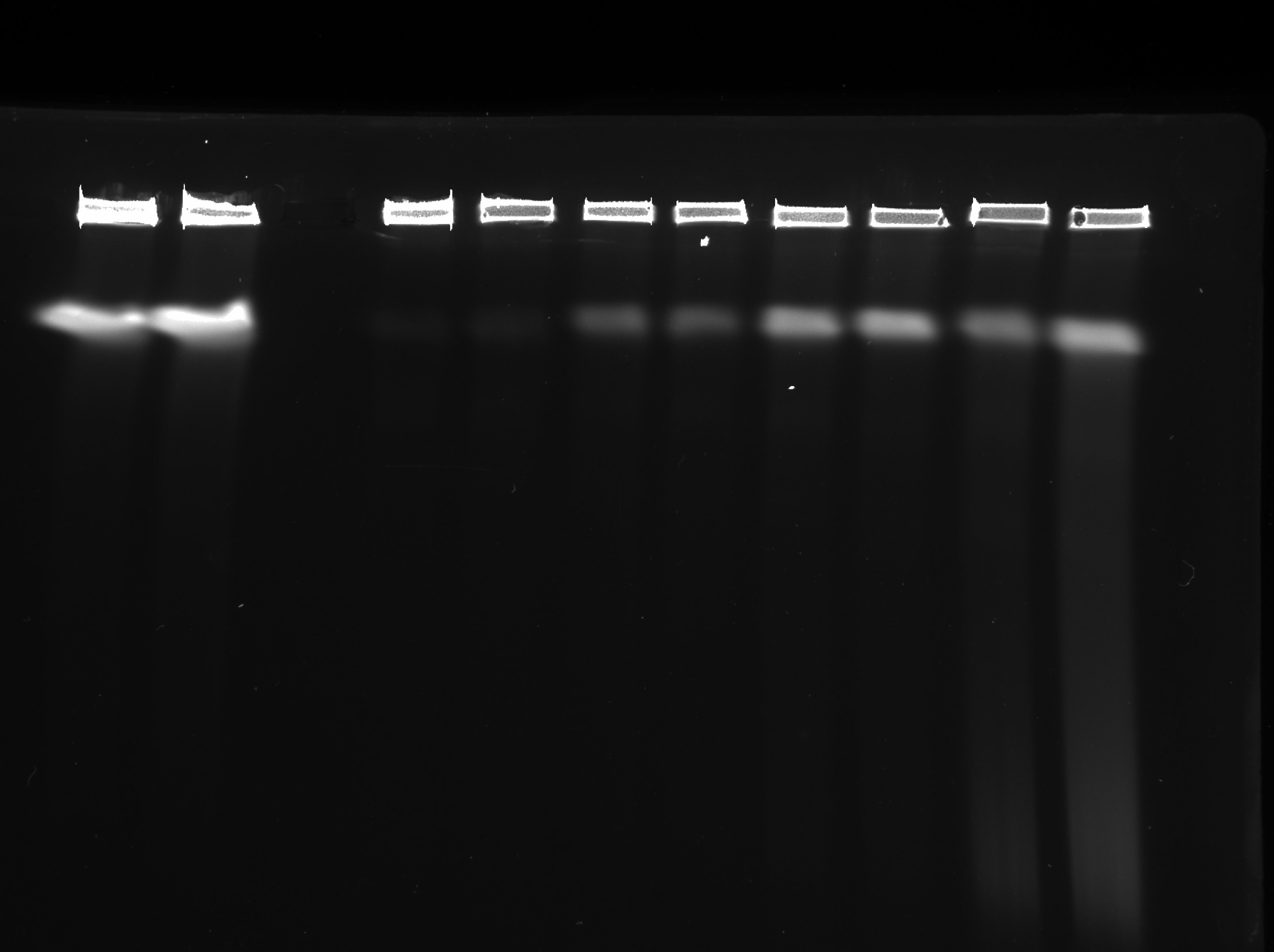

Supplement: Figure 5—figure supplement 1—source data 2. [file elife-104718-fig5-figsupp1-data2.zip › Figure 5-figure supplement 1- Source data 2.tif]
