# Supplementary material for: Synergistic effect of inhibiting CHK2 and DNA replication on cancer cell growth
Source: eLife. 2025 Jan 31;13:RP104718. doi: 10.7554/eLife.104718 (PMC11785374; doi:10.7554/eLife.104718)
Supplement: Figure 3—source data 1. [file elife-104718-fig3-data1.zip › Figure 3-Source data 1.pdf]

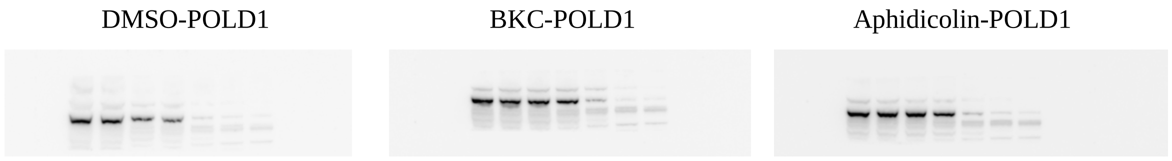

Figure 3, Source Data 1. Original membranes corresponding to Figure 3, panel C.

Figure 3. BKC directly binds to DNA polymerases. A-B. *In silico* molecular docking of bakuchiol in the predicted active site structures of human DNA Pol δ and ε, respectively. **C-D**. MCF-7 cells were treated with DMSO, 40 µM BKC or 10 µM Aphidicolin for 2 hours prior to the cellular thermal sensitivity shift assay (CETSA) at indicated temperature, as described in the Materials and Methods. Levels of POLD1 (Pol δ; panel C) and POLE (Polε; panel D) catalytic subunits were detected by western blotting. Mean and SEM of three independent experiments are shown. The p-values were determined by two-tailed paired t-test.

DMSO-POLE BKC-POLE Aphidicolin-POLE

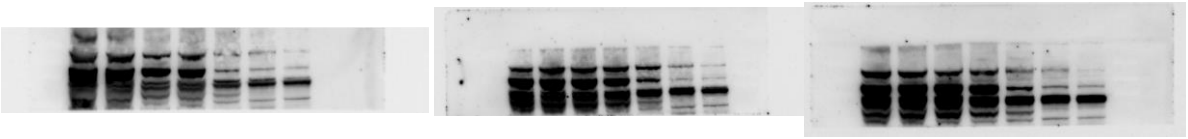

Figure 3, Source Data 1. Original membranes corresponding to Figure 3, panel D.

Figure 3. BKC directly binds to DNA polymerases. A-B. *In silico* molecular docking of bakuchiol in the predicted active site structures of human DNA Pol δ and ε, respectively. **C-D**. MCF-7 cells were treated with DMSO, 40 µM BKC or 10 µM Aphidicolin for 2 hours prior to the cellular thermal sensitivity shift assay (CETSA) at indicated temperature, as described in the Materials and Methods. Levels of POLD1 (Pol δ; panel C) and POLE (Polε; panel D) catalytic subunits were detected by western blotting. Mean and SEM of three independent experiments are shown. The p-values were determined by two-tailed paired t-test.
