# Supplementary material for: Synergistic effect of inhibiting CHK2 and DNA replication on cancer cell growth
Source: eLife. 2025 Jan 31;13:RP104718. doi: 10.7554/eLife.104718 (PMC11785374; doi:10.7554/eLife.104718)
Supplement: Figure 3—figure supplement 1—source data 1. — The treatment and experimental temperatures are indicated. [file elife-104718-fig3-figsupp1-data1.zip › Figure 3-figure supplement 1-Source data 1.pdf]

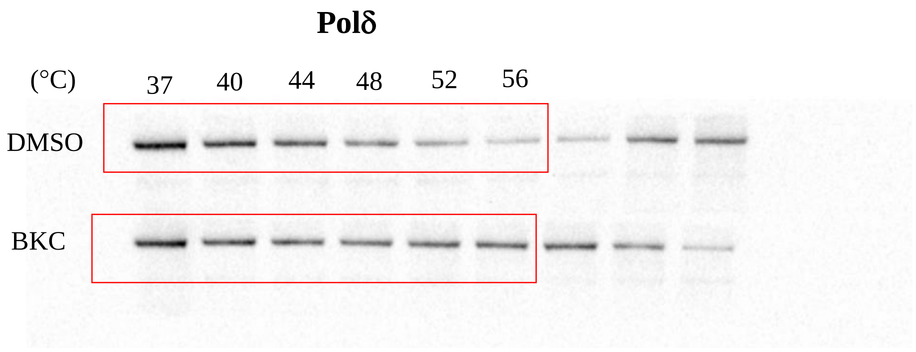

Figure 3-figure supplement 1, Source Data 1. Original membranes corresponding to Figure 3-figure supplement 1, panel A.
The treatment and experimental temperatures are indicated.

Figure 3-figure supplement 1. BKC does not interact with PCNA. A-C. *Xenopus* egg extracts were incubated with DMSO or 40 μM BKC for 2 hours prior to the cellular thermal sensitivity shift assay (CETSA) at indicated temperature, as described in the Materials and Methods. The residual amount of the DNA Polδ catalytic subunit POLD1 (p125, **A**) and the accessory subunit POLD3 (p66, **B**) or PCNA (**C**) in the supernatant was detected by western blotting. Mean of two independent experiments is shown.

POLD3

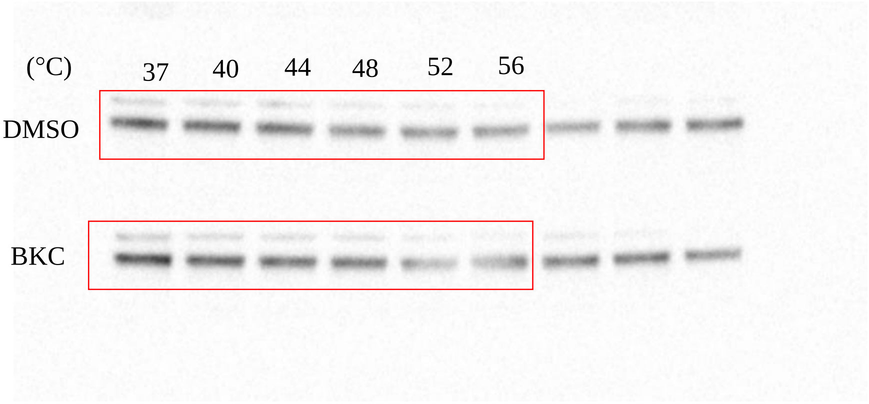

Figure 3-figure supplement 1, Source Data 1. Original membranes corresponding to Figure 3-figure supplement 1, panel B.
The treatment and experimental temperatures are indicated.

Figure 3-figure supplement 1. BKC does not interact with PCNA. A-C. *Xenopus* egg extracts were incubated with DMSO or 40 μM BKC for 2 hours prior to the cellular thermal sensitivity shift assay (CETSA) at indicated temperature, as described in the Materials and Methods. The residual amount of the DNA Polδ catalytic subunit POLD1 (p125, **A**) and the accessory subunit POLD3 (p66, **B**) or PCNA (**C**) in the supernatant was detected by western blotting. Mean of two independent experiments is shown.

PCNA

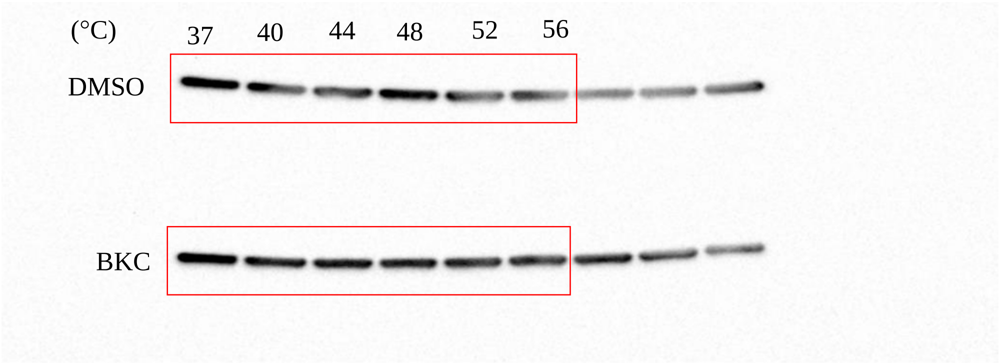

Figure 3-figure supplement 1, Source Data 1. Original membranes corresponding to Figure 3-figure supplement 1, panel C.
The treatment and experimental temperatures are indicated.

Figure 3-figure supplement 1. BKC does not interact with PCNA. A-C. *Xenopus* egg extracts were incubated with DMSO or 40 μM BKC for 2 hours prior to the cellular thermal sensitivity shift assay (CETSA) at indicated temperature, as described in the Materials and Methods. The residual amount of the DNA Polδ catalytic subunit POLD1 (p125, **A**) and the accessory subunit POLD3 (p66, **B**) or PCNA (**C**) in the supernatant was detected by western blotting. Mean of two independent experiments is shown.
