# Supplementary material for: Synergistic effect of inhibiting CHK2 and DNA replication on cancer cell growth
Source: eLife. 2025 Jan 31;13:RP104718. doi: 10.7554/eLife.104718 (PMC11785374; doi:10.7554/eLife.104718)
Supplement: Figure 4—source data 1. [file elife-104718-fig4-data1.zip › Figure 4-Source Data 1.pdf]

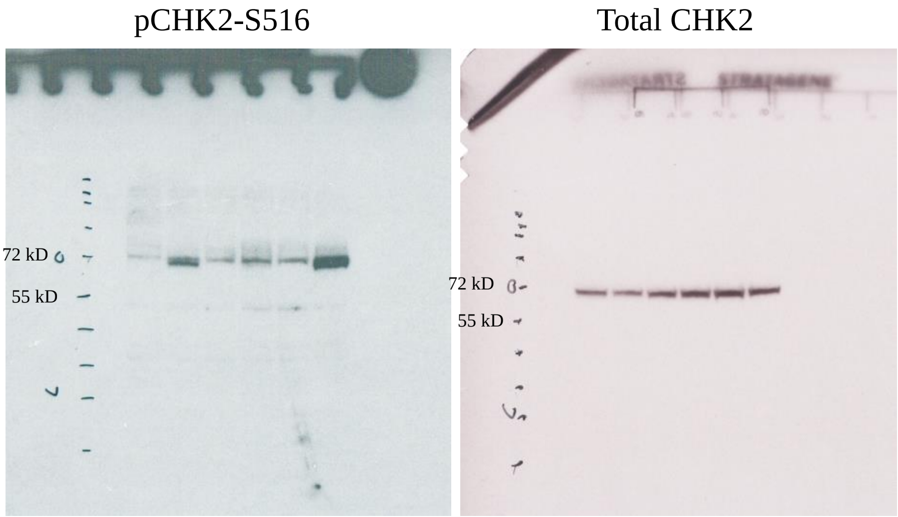

Figure 4, Source Data 1. Original membranes corresponding to Figure 4, panel B. The molecular weight markers are indicated.

Figure 4. IBC inhibits the CHK2 kinase. B. MCF-7 cells were pre-treated with DMSO, 15 µM IBC, or 20 µM BML-277 for 2 hours, then camptothecin (CPT, 1 µM) was added for 2 hours. Phosphorylation of CHK2 on S516 (pCHK2) was detected by western blotting. The ratio of pCHK2-S516 induction, relative to the DMSO+CPT control, is indicated. (n=3)

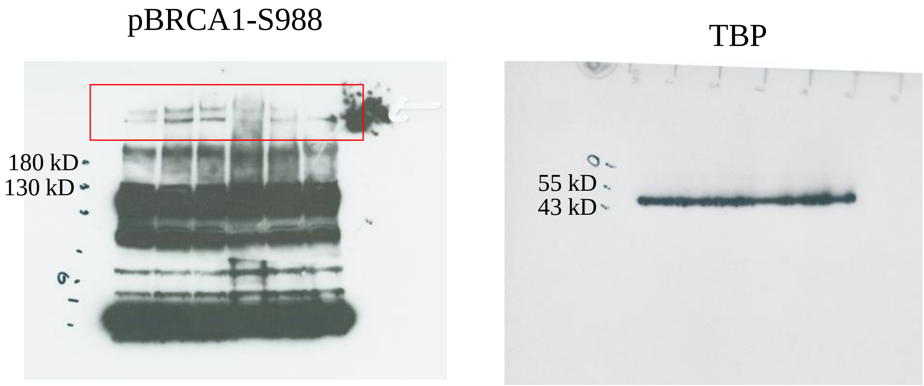

Figure 4, Source Data 1. Original membranes corresponding to Figure 4, panel C. The molecular weight markers are indicated.

Figure 4. IBC inhibits the CHK2 kinase. C. MCF-7 cells were treated as indicated in panel B. Phosphorylation of chromatin-bound BRCA1 at residue S988 was detected by western blotting. The relative ratio of pBRCA1-S988 signal, after normalization to ponceau signal, is indicated. TBP was used as a marker of chromatin fraction

pCHK1-S296

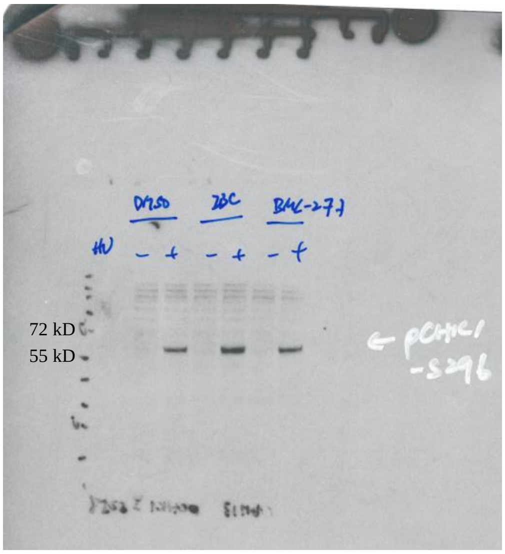

Total CHK1

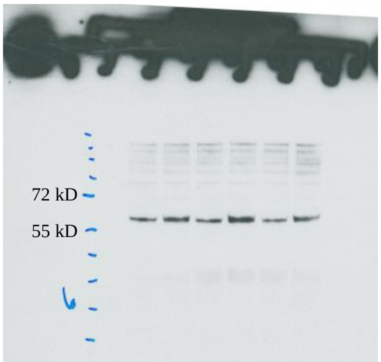

Figure 4, Source Data 1. Original membranes corresponding to Figure 4, panel D. The molecular weight markers are indicated.

Figure 4. IBC inhibits the CHK2 kinase. D. MCF-7 cells were treated with 15 μM IBC for 2 hours, then 4 mM HU was added for 2 hours. CHK1 auto-phosphorylation on S296 (pCHK1) was detected by western blotting.

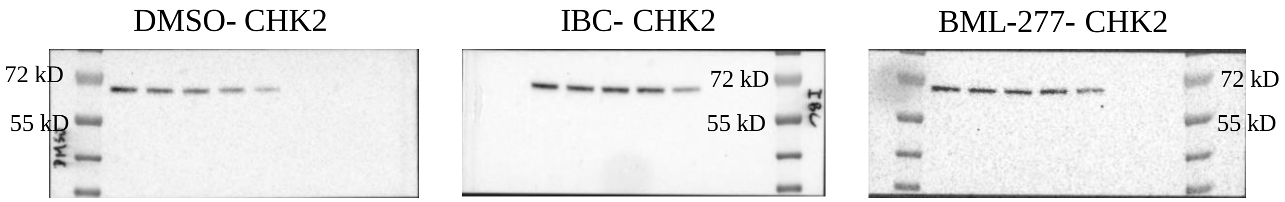

Figure 4, Source Data 1. Original membranes corresponding to Figure 4, panel G. The molecular weight markers are indicated.

Figure 4. IBC inhibits the CHK2 kinase G-H. Cellular Thermal Shift Assay (CETSA) of IBC on the thermal stability of CHK2 and CHK1. MCF-7 cells were treated with 15 µM IBC or 20 µM BML-277 for 2 hours. Cells were proceeded to CETSA as described in the Materials and Methods. The amount of CHK2 and CHK1 present in the supernatant was detected by western blotting. The relative CHK2 and CHK1 signal was quantified. The p-values were determined by two-tailed paired t-test (n=3).

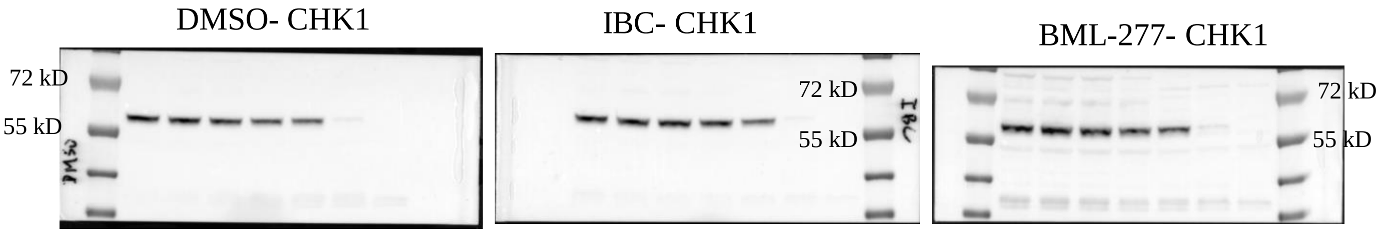

Figure 4, Source Data 1. Original membranes corresponding to Figure 4, panel H. The molecular weight markers are indicated.

Figure 4. IBC inhibits the CHK2 kinase. G-H. Cellular Thermal Shift Assay (CETSA) of IBC on the thermal stability of CHK2 and CHK1. MCF-7 cells were treated with 15 µM IBC or 20 µM BML-277 for 2 hours. Cells were proceeded to CETSA as described in the Materials and Methods. The amount of CHK2 and CHK1 present in the supernatant was detected by western blotting. The relative CHK2 and CHK1 signal was quantified. The p-values were determined by two-tailed paired t-test (n=3).
