# Supplementary material for: Synergistic effect of inhibiting CHK2 and DNA replication on cancer cell growth
Source: eLife. 2025 Jan 31;13:RP104718. doi: 10.7554/eLife.104718 (PMC11785374; doi:10.7554/eLife.104718)
Supplement: Figure 4—figure supplement 1—source data 1. — 4 and 7 are our codes for BKC and IBC, respectively. d: DMSO control; 7+4: means BKC + IBC. [file elife-104718-fig4-figsupp1-data1.zip › Figure 4-figure supplement 1-Source data 1.pdf]

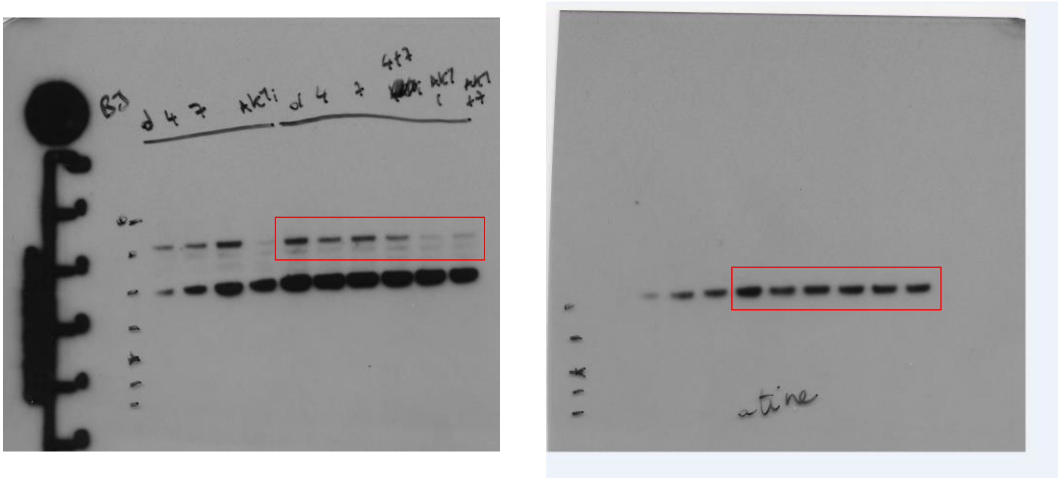

Figure 4-figure supplement 1, Sources Data 1. Original membranes corresponding to Figure 4-figure supplement 1. 4 and 7 are our codes
BKC and IBC, respectively. d: DMSO control; 7+4: means BKC+IBC.

Figure 4-figure supplement 1. IBC does not inhibit AKT activity in MCF-7 cells. A. MCF-7 cells treated with DMSO, 15 μM IBC, 20 μM BKC, the combination IBC + BKC or AKT inhibitor, MK-2206, (AKTi, 10 μM) for 24 hours. Auto-phosphorylation of AKT was detected by immunoblotting analysis. Densitometric quantification of phosphor-AKT signal is shown. (n=2)
