# Supplementary material for: Synergistic effect of inhibiting CHK2 and DNA replication on cancer cell growth
Source: eLife. 2025 Jan 31;13:RP104718. doi: 10.7554/eLife.104718 (PMC11785374; doi:10.7554/eLife.104718)
Supplement: Figure 5—source data 1. — Cytosol for cytosol fraction. Chr for chromatin fraction. Alpha-actin was used as a marker of cytosolic fraction. TBP was employed as a marker of chromatin fraction. The molecular weight markers are indicated. [file elife-104718-fig5-data1.zip › Figure5-Source Data 1.pdf]

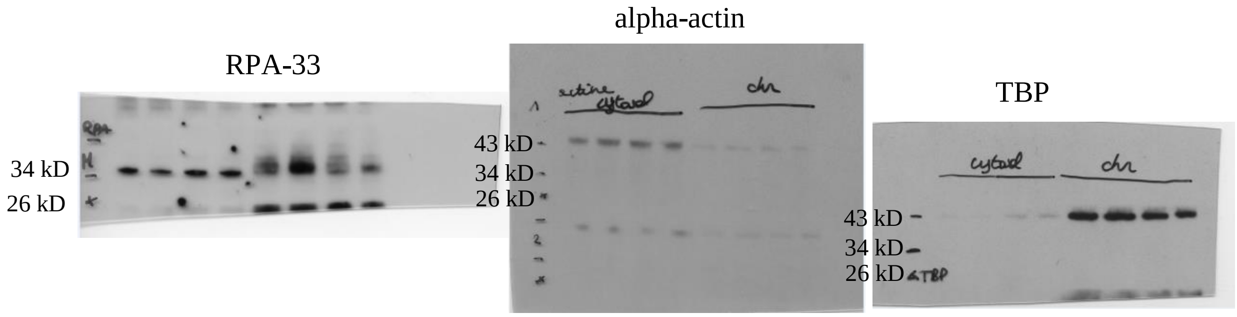

Figure 5, Source Data 1. Original membranes corresponding to Figure 5, panel C. Cytosol for cytosol fraction. Chr for chromatin fraction. Alpha-actin was used as a marker of cytosolic fraction. TBP was employed as a marker of chromatin fraction. The molecular weight markers are indicated.

Figure 5. IBC impedes DNA end resection and RAD51 foci formation. C. MCF-7 cells were treated with DMSO or 15 μM IBC for 2 hours, then camptothecin (CPT, 1 μM) was added for 2 hours, as indicated. Cells were fractionated into cytosol and nuclei (chromatin) and RPA in both fractions was detected by western blotting. Actin and TBP were used as markers of cytosol and chromatin fractions, respectively. The fold change of the chromatin-bound RPA signal relative to the DMSO control was quantified. The p-value was determined by unpaired t-test. (n=3)
