# Supplementary material for: Synergistic effect of inhibiting CHK2 and DNA replication on cancer cell growth
Source: eLife. 2025 Jan 31;13:RP104718. doi: 10.7554/eLife.104718 (PMC11785374; doi:10.7554/eLife.104718)
Supplement: Figure 5—figure supplement 1—source data 1. — Bleocin-treated positive control was included. [file elife-104718-fig5-figsupp1-data1.zip › Figure 5-figure supplement 1-Source data 1.pdf]

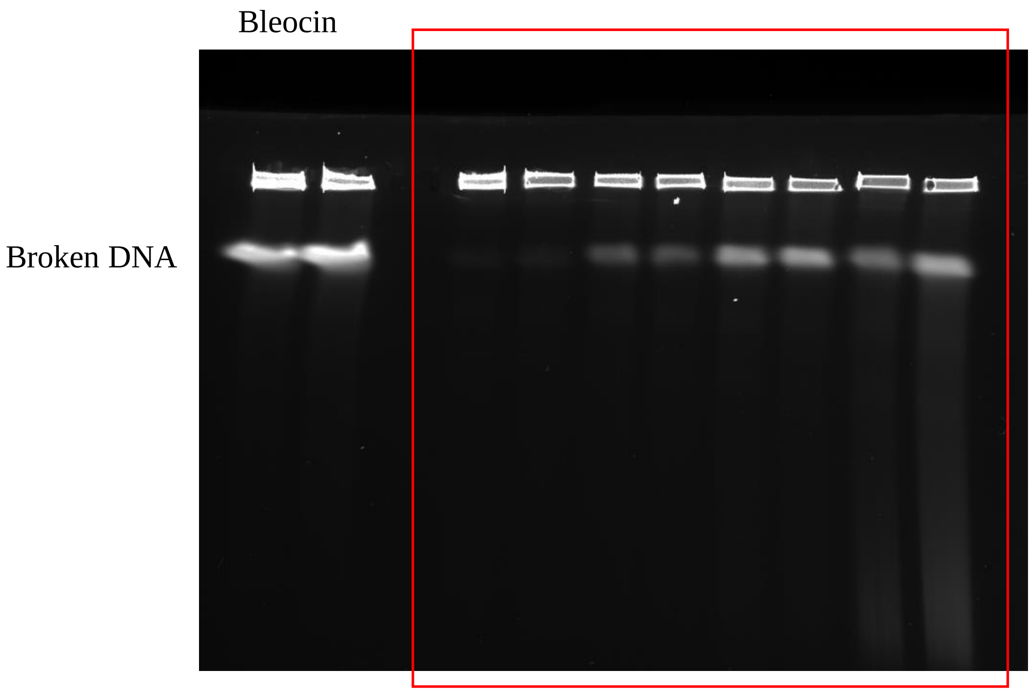

Figure 5-figure supplement 1, Source Data 1. Original gel corresponding to Figure 53-figure supplement 1, panel A.
Bleocin-treated positive control was included

Figure 5-figure supplement 1. IBC results in the persistence of DNA breaks and impairs DNA end resection. A. MCF-7 cells were pre-treated with DMSO or IBC for 1 hour, followed by incubation with or without camptothecin (CPT, 1 µM) for another 2 hours. Cells were recovered immediately or washed and let recover in the presence of DMSO or IBC for 24 or 48 hours. The amount of broken DNA was detected by PFGE. Fold increase of broken DNA was normalized by the control sample at t0. The *p*-values are indicated (two-tailed paired *t*-test). Representative gel image from 4 independent experiments is shown.
